# Branch site bulge conformations in domain 6 determine functional sugar puckers in group II intron splicing

**DOI:** 10.1093/nar/gkz965

**Published:** 2019-10-29

**Authors:** Raphael Plangger, Michael Andreas Juen, Thomas Philipp Hoernes, Felix Nußbaumer, Johannes Kremser, Elisabeth Strebitzer, David Klingler, Kevin Erharter, Martin Tollinger, Matthias David Erlacher, Christoph Kreutz

**Affiliations:** 1 Institute of Organic Chemistry and Center for Molecular Biosciences Innsbruck (CMBI), University of Innsbruck, Innrain 80/82, 6020 Innsbruck, Austria; 2 Institute of Genomics and RNomics, Biocenter, Medical University of Innsbruck, Innrain 80/82, 6020 Innsbruck, Austria

## Abstract

Although group II intron ribozymes are intensively studied the question how structural dynamics affects splicing catalysis has remained elusive. We report for the first time that the group II intron domain 6 exists in a secondary structure equilibrium between a single- and a two-nucleotide bulge conformation, which is directly linked to a switch between sugar puckers of the branch site adenosine. Our study determined a functional sugar pucker equilibrium between the transesterification active C2′-endo conformation of the branch site adenosine in the 1nt bulge and an inactive C3′-endo state in the 2nt bulge fold, allowing the group II intron to switch its activity from the branching to the exon ligation step. Our detailed NMR spectroscopic investigation identified magnesium (II) ions and the branching reaction as regulators of the equilibrium populations. The tuneable secondary structure/sugar pucker equilibrium supports a conformational selection mechanism to up- and downregulate catalytically active and inactive states of the branch site adenosine to orchestrate the multi-step splicing process. The conformational dynamics of group II intron domain 6 is also proposed to be a key aspect for the directionality selection in reversible splicing.

## INTRODUCTION

**Figure 1.**
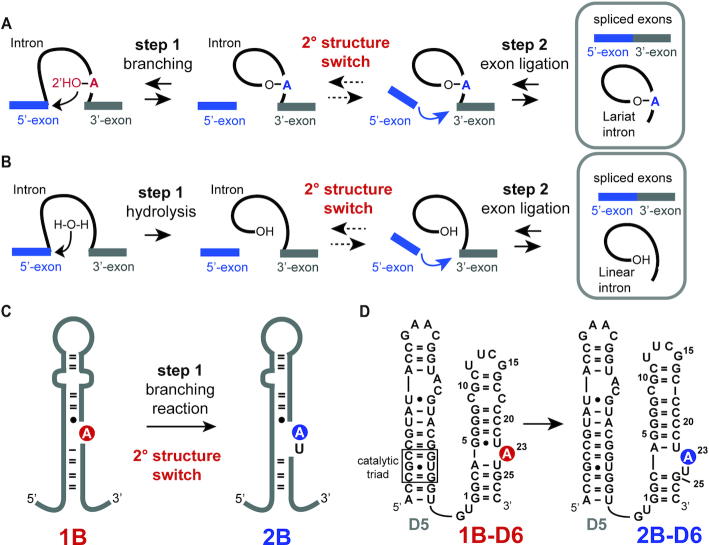
Group II intron splicing pathways, conformational rearrangement between step 1 and 2, D56 construct used in this study. (**A**) Branching pathway: Between step 1 and 2 a secondary structure switch is postulated by Costa and co-workers (16). (**B**) Hydrolysis pathway: Instead of the branch site adenosine a water molecule acts as the nucleophile in the first step of splicing. (**C**) Schematic secondary structure representation of domain 6 of the chimeric Oc19 construct, in which the branching pathway is activated. A rearrangement from a single-bulge (1B) to a two-bulge (2B) between the branching step and the exon ligation step was postulated by Costa *et al.* (16). Branch-site adenosine is highlighted in red and blue. (**D**) *Trans*-active D56 RNA in the 1B and 2B fold state with numbering scheme. Branch-site adenosine is highlighted in red and blue.

Group II introns are catalytically active RNAs with self-splicing activity (1,2). In the branching pathway, a lariat RNA is formed, which resembles lariats formed during splicing of nuclear immature mRNA. This similarity in the reaction pathway suggests that the spliceosome might originate from the older group II introns further supported by an akin structure of domain 5 (D5) in group II introns and the U6/U2 snRNA. Group II introns take influence on gene expression in all domains of life and significant efforts were undertaken to get detailed insights into the chemistry of the ribozyme. X-ray crystallographic studies gave rich information on the intricate three-dimensional architecture of group II introns and the key residues- the conserved catalytic C358-G359-C360 triad and the 2-nucleotide (nt) A376-C377 bulge in D5 and the A287-G288-C289 J2/3 junction along with two catalytically important divalent metal ions (3) – for the second exon ligation step were identified (4–15). Recently, Costa and co-workers succeeded to crystallize a chimeric *Oceanobacillus iheyenis/Azotobacter vinelandii* (Oc19) group II intron from the IIC subclass, in which the branching and not the hydrolysis pathway was activated (Figure 1A and B) (16,17). The lariat intron was obtained and the X-ray structure gave interesting insights into the branch site arrangement and thus into the first step of group II intron catalysis. Based on extensive comparative analysis the branch-site adenosine (A) in domain 6 (D6) of the group II intron was presumed to form a 1nt bulge structure (Figure 1C, fold 1B) (18,19). In the recent crystal structure of Costa and co-workers, however, a basal 3-base pair (bp) stem with the branch point A in a 2nt bulge was consistent with the electron density maps (Figure 1C, fold 2B). Such a 2nt bulge arrangement in D6 was earlier reported in an X-ray crystal structure of a *trans*-active 70 nucleotide D56 construct of the ai5γ group II intron (20). The 2nt bulge is speculated to be the favoured conformational arrangement to promote the second step, the exon ligation. The structure and dynamics of domain 6 and the branching site adenosine were further studied by solution NMR spectroscopy with the main focus on the metal ion binding behaviour (21–23). These studies, however, used D6 motifs focusing mainly on the branch site A containing bulge with shortened stems and thus a crucial aspect in the dynamic behaviour of domain 6 was overlooked. Using NMR spectroscopy, we addressed the secondary structure rearrangement in a group II intron D56 construct (Figure 1D). In a 61nt D56 RNA, an associated secondary structure and sugar pucker equilibrium was observed, which was found again in several isolated D6 constructs from other group II intron subclasses. The reversible secondary structure/sugar pucker equilibrium in D6 unveiled by NMR spectroscopy supports a conformational selection mechanism in group II intron splicing. The tuneable conformational equilibrium, which can be influenced by external cues, such as magnesium ions, serves as a conformational pool to guarantee the availability of active and inactive states during the multistep catalysis and also to switch the directionality of the splicing process.

## MATERIALS AND METHODS

### Sample preparation

Standard 2′-*O*-TBDMS RNA phosphoramidites (rA^Ac^, rC^Ac^, rG^Ac^ and rU, *Chemgenes*, USA) were used in combination with in-house synthesized ^13^C-, ^15^N- and ^2^H-modified RNA phosphoramidites. The synthetic access to the stable isotope labelled phosphoramidites was earlier described (24–27). Controlled pore glass (CPG) RNA solid support (1000 Å pore size, *ChemGenes*, USA) with an average loading of 40 μmol g^−1^ was used to synthesize the RNAs on an ABI 391 PCR Mate using a self-written RNA synthesis cycle. Amidite (0.1 M) and activator (5-benzylthio-*1H*-tetrazole, 0.25 M) solutions were dried over freshly activated molecular sieves (3 Å) for at least 48 h. The following reagent mixtures were used: *Cap A*: acetic anhydride/lutidine/tetrahydrofuran 1/1/8 (v/v/v). *Cap B*: tetrahydrofuran/*N*-methylimidazole 86/16 (v/v). *Oxidation solution*: 500 mg iodine dissolved in a mixture of 70 ml THF, 20 ml pyridine and 10 ml water. *Detritylation solution*: 4% dichloroacetic acid in anhydrous toluene. After complete RNA synthesis, the solid support was dried in 1 h in high vacuum. *Standard alkaline deprotection*: 650 μl aq. methylamine solution (40%) and 650 μl aq. ammonia solution (28–30%) were added to the solid support. The reaction tube was shaken vigorously and incubated at 37°C for 6 h. The solid support was pelleted via centrifugation and the supernatant was transferred to a 10 ml round bottom flask. The remaining solid support was washed three times with a mixture of THF/water (1/1), the liquid phases were combined with the first filtrate and evaporated to dryness. The residual white precipitate was dried in high vacuum for at least 1 h. *2′-O-TBDMS deprotection:* The residue from the previous step was dissolved in 300 μl anhydrous dimethylsulfoxide, and triethylamine trihydrofluoride was added (50 eq. per TBDMS group) and the deprotection mixture incubated at 37°C for at least 16 h. Then, the deprotection mixture was quenched with 2 ml quenching buffer (*GlenResearch*, USA) and directly applied to a HiPrep 26/10 desalting column (*GE Healthcare*, Austria) using a ÄKTA start system (*GE Healthcare*, Austria). The crude RNA was eluted using HPLC grade water and the RNA containing fractions (UV detection at 254 nm) were collected in a 50 ml round bottom flask. After evaporation, the crude RNA was dissolved in 1 ml HPLC grade water and transferred to a 1.5 ml reaction tube. The crude RNA was stored at −20°C. The quality of the crude RNAs was checked via anion exchange chromatography on an analytical Dionex DNAPac PA-200 column (4 × 250 mm; *Eluent A*: 25 mM Tris−HCl, 6 M urea, pH 8.0; *Eluent B*: 25 mM Tris−HCl, 500 mM sodium perchlorate, 6 M urea, pH 8.0) and at elevated temperature (80°C). Purification of the RNA sequences was achieved in a single run by applying the crude RNA on a preparative Dionex DNAPac PA-200 column (22 × 250 mm, eluents as before). The fractions containing the desired RNA were pooled and loaded on a C18 SepPak catridge (*Waters*, Austria) to remove HPLC buffer salts. The RNA sodium salt form was then eluted from the C18 column with water/acetonitrile (1/1, v/v), concentrated and transferred to a 1.5 ml reaction tube for concentration determination and mass spectrometric analysis. Sample concentrations were determined by measuring UV absorption at 260 nm on a NanoPhotometer (*Implen*, Germany).

### Synthetic access to the 2′-photolabile protected adenosine building block

The photolabile α-methyl-*o*-nitrophenyl (NPE) protecting group was attached to the 2′-hydroxyl of adenosine, which was then transformed into a phosphoramidite building block suitable for solid phase RNA synthesis. In the first step, the NPE group was attached to adenosine 2′-OH and 3′-OH under alkaline conditions. The resulting mixture of 2′- and 3′-substituted products was successfully separated via silica column chromatography after the introduction of a phenoxyacetyl moiety at the exocyclic amino group. Subsequently, the 2′-and *N*^6^-protected nucleoside was transformed to its 5′-*O*-4,4′-dimethoxytrityl derivative under standard conditions before the 2-cyanoethyl-*N*,*N*-diisopropyl phosphoramidite function was introduced to yield the 2′-*O*-photolabile protected adenosine building block 4 for RNA solid phase synthesis. A detailed description for the preparation can be found in the supplementary information (Supplementary Scheme S1).

### Synthesis of the branched RNA mimic

For branched RNA synthesis, the 2′-photolabile NPE protected adenosine building block was used in combination with standard RNA phosphoramidites as described in the sample preparation section. Prior to the attachment of nucleotides at the 2′-oxygen of the branch site A23, the 2-cyanoethyl protection groups of the phosphodiester backbone were removed from the solid support bound RNA with 10% diethylamine in acetonitrile (20 ml with a flowrate of 3 ml min^−1^), followed by washing with 50 ml acetonitrile and drying the solid support in high vacuum for 1h. This step is crucial to avoid a strand cleavage after the removal of the photolabile NPE protecting group. The solid support bound RNA was suspended in 2 ml methanol in a UV cuvette and constantly shaken while exposed to UV light (wavelength 260 nm) for 5 h at room temperature. Then, the solid support was washed 3× with 5 ml methanol, and 3× with 10 ml dry acetonitrile, before it was again connected to the RNA synthesizer. The additional nucleotides were added to the A23 2′-oxygen using commercially available reverse direction RNA phosphoramidites (*Chemgenes*, USA) giving the desire 2′-5′-phosphodiester linkage in the branched RNA. Concentrated reverse amidite solutions (0.3 M) were dried over freshly activated molecular sieves (3 Å) for at least 48 h. The coupling time for the reverse amidites was increased to 8 min. For capping, oxidation and deblocking the standard reagents described in the sample preparation section were used. The branched RNA was deprotected and purified according to the previously described protocol.

### RNA LC–MS analysis

All RNAs were analyzed on Finnigan LCQ Advantage MAX ion trap instrumentation connected to a Thermo Scientific UHPLC (components: Ultimate 3000 RS Pump, Ultimate 3000 RS Autosampler, Ultimate 3000 RS Column Compartment, Ultimate 3000 Diode Array Detector). RNA mass spectra were acquired in the negative-ion mode with a potential of −4 kV applied to the spray needle (capillary temperature: 270°C, capillary voltage: −2−3 V). LC: 250 pmol RNA dissolved in 30 μl of 20 mM ethylenediaminetetraacetic acid (EDTA) solution; average injection volume: 30 μl; column: Waters xBridge C18 2.5 μm column (1.0 × 50 mm) at 30°C; flow rate: 100 μl/min; eluent A: 8.6 mM triethylamine (TEA), 100 mM 1,1,1,3,3,3-hexafluoroisopropanol in H_2_O (pH 8.0); eluent B: methanol; gradient: 0–100% B in A within 30 min; UV detection at 260/280 nm. The correct assembly of all RNAs used in this study was confirmed by the mass data.

### NMR spectroscopy

RNA samples were lyophilized as sodium salts and dissolved in 280 μl NMR buffer (15 mM sodium phosphate, 25 mM NaCl, 0.1% NaN_3_, pH 6.9) and transferred into restricted volume *Shigemi* tubes giving 0.7–1 mM sample concentrations. NMR experiments on ^13^C- and ^15^N-modified RNA sequences were conducted either on a Bruker 600 MHz Avance II+ NMR with a Prodigy TCI probe or on Bruker 700 MHz Avance Neo system with a QXI (^1^H/^13^C/^15^N/^31^P) room temperature probe. The 2D ^1^H–^13^C correlation spectra were acquired using either a ^1^H–^13^C-BEST TROSY pulse sequence or a standard HMQC Bruker pulse program (*hmqcetgpsi.2*). The 2D ^1^H–^15^N correlation spectra were acquired using a ^1^H–^15^N-SOFAST HMQC pulse sequence. The fold-specific Watson Crick base pairs were determined using a HNN–COSY experiment from the Bruker pulse sequence library (*na_hnncosygpphspwg*). The ^13^C-longitudinal exchange data for the D56 ^13^C1′-modified RNA was determined using an alternative approach combining a T_1_ and a longitudinal exchange NMR experiment (28). Arrays of T_1_ and ZZ exchange spectra were recorded at 14.1 T at 25°C with mixing times of 5, 10, 20, 30, 40, 50, 60, 70, 80, 90 and 100 ms. The size of the data matrices for each spectrum was 1024*96 complex data points, the number of scans was 96 and the inter-scan delay was 1.25 s to yield a total measuring time of 84 h for eleven T_1_ experiments (42h) and eleven ZZ exchange (42 h) experiments, respectively.

### ^13^C longitudinal exchange data fitting

Peak intensities at the various mixing periods were obtained by summing over 1 × 1 (f_1_ × f_2_) data points using the nmrpipe software package (29). Then, a least square fitting procedure was applied to determine *k*_1B→2B_, *k*_2B→1B_ and R_1_^1B^ and R_1_^2B^ by fitting the following expressions in MATLAB:

$$\begin{eqnarray*} {M_{AA}}(t)/{M_{AA}}(0) = {1 \over {({\lambda _1} - {\lambda _2})}}[({a_{22}} + {\lambda _1}){e^{{\lambda _1}t}} - ({a_{11}} + {\lambda _2}){e^{{\lambda _2}t}}] \end{eqnarray*}$$

$$\begin{eqnarray*} {M_{BB}}(t)/{M_{BB}}(0) = {1 \over {({\lambda _1} - {\lambda _2})}}[({a_{11}} + {\lambda _1}){e^{{\lambda _1}t}} - ({a_{22}} + {\lambda _2}){e^{{\lambda _2}t}}] \end{eqnarray*}$$

and

$$\begin{eqnarray*} {M_A}\left( t \right)/{M_A}\left( 0 \right) &=& \frac{1}{{\left( {{\lambda _1} - {\lambda _2}} \right)}}\\ &&\times \left[ {({a_{22}} - {a_{21}} + {\lambda _1}} \right){e^{{\lambda _1}t}} - ({a_{22}} - {{\rm{a}}_{21}} + {\lambda _2}){e^{{\lambda _2}t}}] \end{eqnarray*}$$

$$\begin{eqnarray*} {M_B}\left( t \right)/{M_B}\left( 0 \right) &=& \frac{1}{{\left( {{\lambda _1} - {\lambda _2}} \right)}}\\ &&\times \left[ {({a_{11}} - {a_{12}} + {\lambda _1}} \right){e^{{\lambda _1}t}} - ({a_{11}} - {{\rm{a}}_{12}} + {\lambda _2}){e^{{\lambda _2}t}}] \end{eqnarray*}$$

with *M_AA_* and *M*_*BB*_ are the intensities of the correlation peaks from the ZZ exchange experiment. These intensities are normalized by the peak volumes at zero mixing time (*M_AA_*^0^ and *M*_*BB*_^0^). *M_A_*(0)/*M_B_*(0) and *M*_*A*_(*t*)/*M_B_*(*t*) are the peak intensities of fold A and B at zero mixing time or the mixing time *t* from the T_1_ experiment. The eigenvalues of the spin density matrix with magnetization transfer effects from chemical exchange are given by

$$\begin{eqnarray*} {\lambda _{1/2}} = \frac{1}{2}[ - ({a_{11}} + {a_{22}}) \pm {\left[ {{{\left( {{a_{11}} - {a_{22}}} \right)}^2} + 4{a_{12}}{a_{21}}} \right]^{1/2}} \end{eqnarray*}$$

with the elements *a_ij_* defined as:

$$\begin{eqnarray*} {a_{11}} &=& {k_{1B \to 2B}}+ R_1^{1B},{a_{22}} = {k_{2B \to 1B}}+ R_1^{2B},{a_{12}}\\ &=& - {k_{2B \to 1B}}\ \ {a_{11}} = {k_{1B \to 2B}}\ \end{eqnarray*}$$

Parameters *k*_1*B*→2*B*_ and *k*_2*B*→1*B*_ describe the refolding rate constants describing the interconversion between the two folding states (1B and 2B) of the D56 RNA, while *R*_1_^1*B*^ and *R*_1_^2*B*^ are the longitudinal relaxation rates in conformation 1B and 2B, respectively. Experimental uncertainties were estimated from 1000 Monte Carlo runs in which synthetic data sets were generated from the best-fit values of *k*_1*B*→2*B*_, *k*_2*B*→1*B*_ and *R*_1_^1*B*^, *R*_1_^2*B*^ by adding random errors based on the signal-to-noise ratio to the best-fit curves.

### Sugar pucker determination of U12 in 1B and 2B and A23 in 1B fold

To address the sugar pucker in the 1B and 2B fold we adapted the H(N)HA experiment originally proposed for the determination of the three bond scalar coupling constant between the amide proton and the α-proton in proteins (30). The experiment was run as the ^13^C version—termed H1′(C1′)H2′ experiment—on the two ^13^C1′-labelled D56 RNA mutants. In detail, the experiment was run on 1 mM 1B and 2B mutant RNA samples in restricted volume *Shigemi* tubes in a 2D manner without ^13^C chemical shift evolution. The data matrix for each spectrum was 1024 × 96 complex data points with 12 ppm spectral width in both proton dimension, the number of scans was 384 and the inter-scan delay was 1 s to yield a total measuring time of 12 h per spectrum. The cross peak/diagonal peak ratio of U12 in both folds and A23 in the 1B fold provide a direct measure of the ^3^*J*_H1’H2’_ according to the equation:

$$\begin{eqnarray*}\ \frac{{{S_{cross}}}}{{{S_{diag}}}} = \ - ta{n^2}\left( {2\pi {J_{HH}}\zeta } \right)\end{eqnarray*}$$

with *S*_cross_ crosspeak intensity, *S*_diag_ crosspeak intensity, *J*_HH_ the 3-bond H1′–H2′ scalar coupling constant and the ζ delay set to 13.05 ms. The cross- and diagonal peak intensities were obtained by using the nmrpipe *nlinLS* peak fitting procedure using Lorentzian peak fitting (29).

### Melting curve analysis of the 61nt D56 constructs

The RNAs were lyophilized and then dissolved in 800 μl (1 cm cuvettes) or 300 μl (1 mm cuvettes) melting curve buffer (10 mM sodium phosphate, pH 7.0, 150 mM NaCl) to give RNA concentrations of 2 and 5 μM, respectively. Absorbance versus temperature profiles were recorded at 250 and 260 nm on a Cary-100 spectrophotometer equipped with a multiple cell holder and a Peltier temperature-control device. Data were collected for five heating-cooling cycles at a rate of 0.7°C/minute. Melting transitions were essentially the same with respect to the two different wavelengths and heating-cooling cycles. Melting point temperatures are reported as mean value of the five measurements. The thermodynamic parameters of the monomolecular melting processes of the D56 1B and 2B mutants were obtained by plotting the association degree *α* versus temperature and fitting the experimental data in *KaleidaGraph* (Synergy Software) using the following equation:

$$\begin{eqnarray*}{\rm{\alpha \ }} = \frac{1}{{1 + {e^{\frac{{{\rm{\Delta }}{H^0} - T{\rm{\Delta }}{S^0}}}{{RT}}}}}}\ \end{eqnarray*}$$

with α association degree and *R* ideal gas constant to give the enthalpy Δ*H*° and the entropy Δ*S°* of the melting transition at 298 K. For all RNAs- the wildtype D56 RNA, the 1B mutant RNA and the 2B mutant RNA-concentration independent melting points indicative for monomolecular folds were observed (Supplementary Figures S13, S14 and Table S3).

## RESULTS

### A secondary structure equilibrium in a D56 Oc19 RNA construct with a hierarchical kinetic network

**Figure 2.**
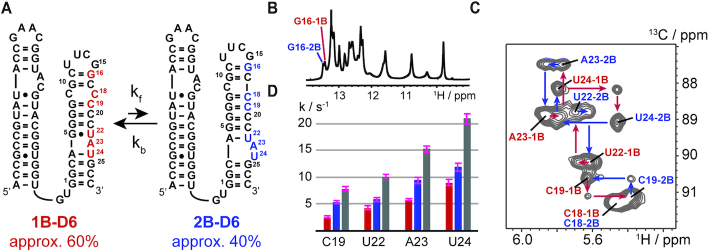
Secondary structure equilibrium in D56. (**A**) The 61nt D56 RNA with ^13^C1′-labels highlighted in red (1nt bulge fold - 1B) and blue (2nt bulge fold - 2B). Equilibrium populations of 1B and 2B are given. (**B**) Imino proton spectrum of the 61nt D56 RNA. The two NH1 resonances for G16 were assigned by site-specific ^15^N^1^-G labeling and confirm the slow exchange process in D6. (**C**) ^13^C longitudinal exchange spectrum with ^13^C1′-labelled C18, C19, U22, A23 and U24 at 25°C and 50 ms mixing time. Assignments are given. (**D**) Exchange rate constants in s^−1^ determined from ^13^C longitudinal exchange NMR experiments. Red forward rate constant *k*_f_, blue backward rate constant *k*_B_ and grey exchange rate *k*_ex_ = *k*_f_ + *k*_b_.

Group II introns are composed of six domains with D5 forming extensive interactions with D1 to shape the catalytic center for the second exon ligation step (Supplementary Figure S1). The branch-point adenosine resides in D6, whose 2′-hydroxyl functionality serves as the nucleophile in the branching reaction. An RNA comprising only D5 and D6 is able to catalyse branched RNA formation and the release of the 5′-exon, when added *in trans* to a D1 RNA (20). Thus, we decided to study the folding behavior of D6 in a D56 sequence spanning a 61-nucleotide region of the group II intron *Oc19* intron using solution NMR spectroscopy (Figure 2a). Five residues—C18, C19, U22, A23 and U24—of the D56 RNA were replaced by the ^13^C-labeled C1′ counterparts. First, we acquired an imino proton spectrum of this D56 RNA and found a complex signal pattern (Figure 2B). Even more surprisingly, instead of five ^1^H–^13^C correlations in a heteronuclear multi quantum correlation (HMQC) spectrum nine peaks with strongly varying intensities were observed (Figure 2C). The resonance assignment procedure revealed that the D56 RNA construct is slowly exchanging between two competing secondary structures—the 1B fold and the 2B fold— on the chemical shift time scale resulting in two resonances for each peak but C18 (Figure 2C).

By making extensive use of ^13^C and ^15^N-isotope labeling via solid phase synthesis the secondary structure equilibrium between the 1B fold with A23 in the bulged conformation and a 4-base pair (bp) basal stem and the 2B fold with a A23-U24 two nucleotide bulge and a 3-bp basal stem could be verified. All the residues, which were replaced by the ^13^C or ^15^N labeled counterparts in the course of this study, are shown in the supporting information (Supplementary Figure S2). For example, the G7-C20 base pair in 1B and the co-existing G7-C19 of 2B could be unambiguously verified by HNN-COSY experiments further confirming the secondary structure equilibrium in D6 (Supplementary Figure S3). The populations of the 1B and 2B fold could be estimated by peak integration and a slight preference for 1B with 60% was found. The secondary structure equilibrium between 1B and 2B was further characterized by ^13^C-longitudinal exchange NMR spectroscopy and a hierarchical kinetic network was found (Figure 2C and D, Supplementary Figure S4 and S5, Supplementary Table S1). The highest exchange rate, *k*_ex_, as the sum of forward (*k*_f_) and backward (*k*_B_) exchange rate, was found for residue U24 with 20.89 ± 0.91 s^−1^, followed by A23 (15.24 ± 0.57 s^−1^) and the exchange rate decreases steadily approaching the cUUCGg tetraloop (U22, *k*_ex_ = 10.11 ± 0.47 s^−1^ and C19, *k*_ex_ = 7.88 ± 0.39 s^−1^). The same rate differentiated kinetics was found for a D56 construct with aromatic nucleobase 6–^13^C- and 8–^13^C-labels on U12, U13, U24, A4 and A23 (Supplementary Figure S4). This hierarchical kinetic network suggests that in both the forward and the backward folding pathway the one or two nucleotide bulge regions, respectively, serve as the starting point for the refolding process. Based on the rate constants, the folding from 1B to 2B is initiated by the formation of the A4–U22 base pair and the concomitant formation of the A23–U24 2nt bulge, followed by the base pair register change of the central G–C rich tract and completed by the base pairing of G8 and C18. In a similar way, refolding from 2B to 1B starts by the formation of the A4–U24 and G5•U22 base pairs again followed by base pair register change of the central G–C rich tract and is finished by the formation of C9-C18 symmetric bulge (Supplementary Figure S6).

Noteworthy, a highly modular structure of the group II intron is confirmed by our NMR data as the secondary structure equilibrium is preserved in a 27nt D6 only construct. Further, chemical shifts of the imino proton and ^1^H–^13^C correlations of D6 residues are virtually identical in the 61nt and 27nt RNA indicating no or very weak tertiary interactions between the two domains. The equilibrium between the 1B- and 2B-fold is found in this shorter D6 RNA with unchanged equilibrium populations and similar kinetics (Supplementary Figure S7, Table S1).

### Shifting the secondary structure equilibrium

**Figure 3.**
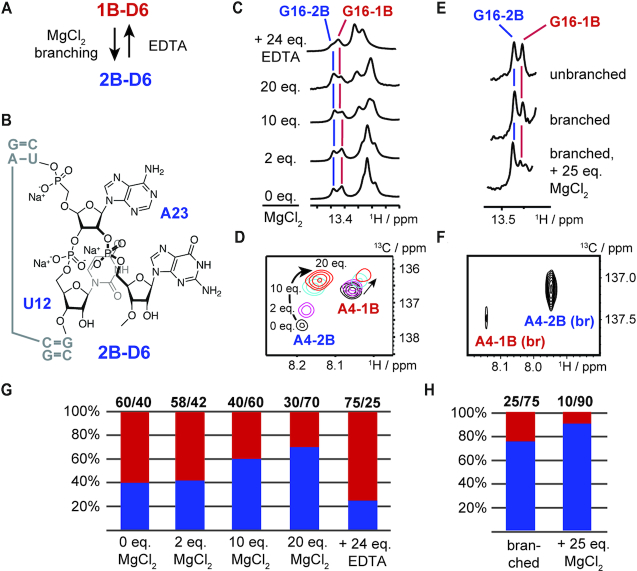
Regulators of the 1B–2B fold equilibrium populations. (**A**) Schematic summary of factors tuning the equilibrium populations. The addition of MgCl_2_ and branched RNA formation lead to a preference of 2B, whereas the addition of ethylenediaminetetraacetic acid (EDTA), a chelating agent for magnesium (II) ions, favours fold 1B. (**B**) Schematic structure of the 2nt bulge in 2B. The 2nt bulge allows a higher degree of conformational flexibility in the branched state. (**C**) G16 N^1^H imino resonance a function of MgCl_2_ and EDTA additions. (**D**) HMQC spectrum of the A4 ^1^H^8^–^13^C^8^-correlation as a function of MgCl_2_ additions. (**E**) G16 N^1^H imino resonance in branched and unbranched state. (**F**) HMQC spectrum of the A4 ^1^H^8^–^13^C^8^-correlation in the branched state with 25 eq. MgCl_2_ added. (**G**) Bar plots of equilibrium populations at various MgCl_2_ and EDTA concentrations. (**H**) Bar plots of equilibrium populations in branched state with and without magnesium (II) ions.

The experiments so far focused on the equilibrium between 1B and 2B in the pre-catalytic state, where the slightly favored 1B-fold is presumed to be the active species in forming the branched RNA. However, during the multi-step catalytic process the group II intron ribozyme must be able to reshuffle the population from 1B towards the 2nt bulge fold, as 2B is speculated to be the active species in the exon ligation step. We thus started to search for triggers switching the equilibrium position in D6 (Figure 3A).

Two magnesium (II) ions are essential to form the active site catalytic triad in D5 and are involved in exon ligation catalysis (15). Thus, we speculated that the addition of magnesium (II) ions might also influence the fold population distribution in D6. Very strikingly, we saw a strong fold shifting effect upon the addition of magnesium (II) chloride. The equilibrium position change was reflected in the intensity change of the G16 N^1^H resonance in the imino proton spectrum (Figure 3C) and also in the ^1^H–^13^C-HMQC spectra of an 8–^13^C A4 labelled D56 RNA (Figure 3D). By using peak integration of the ^1^H8–^13^C8-correlation signal of A4 we found a decrease of fold 1B from 60% to 30% and the corresponding increase from 40% to 70% for 2B (Supplementary Table S2). The equilibrium reshuffling process reached a steady state at 20 eq. of Mg^2+^-ions (15 mM) with respect to the strand molarity (0.75 mM) of the D56 RNA. As the physiological Mg^2+^-ion concentration can be up to 1 mM in cells (31), 20 equivalents of magnesium (II) ions would correspond to a plausible group II intron RNA strand concentration of about 50 μM in a cellular environment. We also probed potential magnesium (II) ion binding sites by running an imino NOESY experiment in the presence of hexamminecobalt (III) chloride, a mimic for hydrated magnesium ions (Supplementary Figure S8). Two binding sites could be identified—a strong NOE to the G6 H1 in fold 2B points towards specific magnesium binding in the central G–C rich tract of 2B and a low-intensity NOE to U6 H3 indicates weak and unspecific cation binding in the UUCG tetraloop. The magnesium ion induced switching is fully reversible as the addition of 24 eq. of ethylenediaminetetraacetic acid (EDTA), a chelating agent for magnesium (II) ions,) with respect to the D56 strand molarity led to a change of the fold equilibrium with an 1B-population of 75%.

Next the folding behaviour of a branched D6 RNA was investigated to probe the influence of the first step – the branching reaction. Using solid phase RNA synthesis an 8–^13^C-A4 modified 27nt *Oc19* D6 RNA was synthesized and relying on a 2′-photolabile protected adenosine building block and reverse amidites an RNA tetramer was attached to the 2′-OH of the branch-site adenosine A23 (Figure 3B, Supplementary Scheme S1, Figure S9) (32). Then, the 1B/2B-fold distribution was estimated from the imino proton region of the NMR spectrum again focusing on the resolved G16 N^1^H resonances and from the ^1^H–^13^C-correlation of the 8–^13^C-A4 in the branched RNA (Figure 3E and F). In the branched RNA, the fold equilibrium is strongly shifted toward the 2B conformation with a population of approximately 75%, which upon the addition of 25 eq. of magnesium (II) ions was almost exclusively pushed towards the 2nt-bulge state (90%). The preference very likely results from the unfavorable steric arrangement in 1B by the attachment of additional nucleotides at the 2′-hydroxyl of A23. The branching puts a high steric pressure at the bulge site in the 1nt-bulge conformation. This steric pressure is counteracted by switching into fold 2B as in this state a structural relief due to the higher conformational flexibility of the 2nt bulge is possible. This leads to a better accommodation of the branched RNA structure within the group II intron RNA and also prepares the ribozyme for the subsequent exon ligation step (Figure 3B). The branching induced equilibrium shift is in accordance with the assumption that 1B is the active conformation in the first branching step. After the formation of the branched lariat, 1B gets unfavored and the population of the state 2B increases up to 90% setting the stage for the subsequent exon-ligation reaction.

### Sugar pucker of the branch-site adenosine A23 in the 1B and 2B

**Figure 4.**
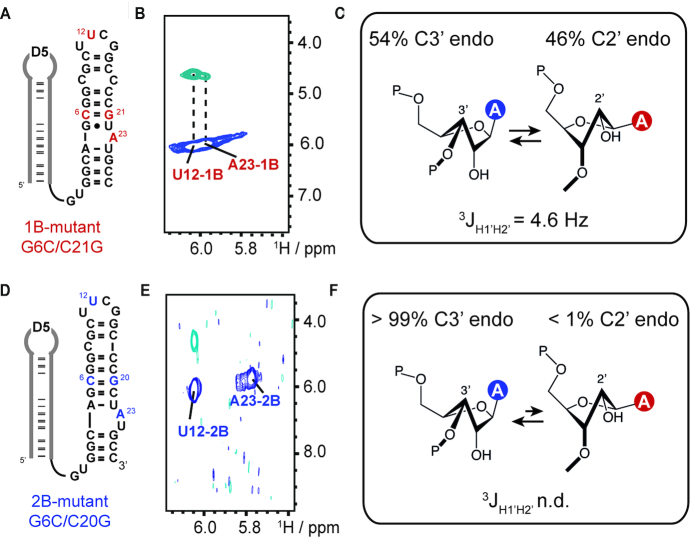
Sugar puckers in 1B and 2B. (**A**) Secondary structure representation of the G6C/C21G mutant populating only state 1B. Mutations are highlighted in red. U12 and A23 also highlighted in red carry a ^13^C1′ label. (**B**) H1′(C1′)H2′ experiment for the determination of the sugar pucker. For both residues a cross peak was observed. (**C**) Schematic representation of sugar pucker equilibrium of A23 in state 1B. A ^3^*J*_H1_′_H2_′ = 4.6 Hz scalar coupling constant was determined for A23 translating in a 54/46 C3′/C2′-endo sugar pucker equilibrium. (**D**) Secondary structure representation of the G6C/C20G mutant populating only state 2B. Mutations are highlighted in blue. U12 and A23 also highlighted in blue carry a ^13^C1′ label. (**E**) H1′(C1′)H2′ experiment for the determination of sugar pucker. Only for U12 a cross peak was observed. (**F**) Schematic representation of sugar pucker equilibrium of A23 in state 2B. The ^3^*J*_H1_′_H2_′ Hz scalar coupling constant could not be determined for A23 translating in a purely C3′-endo sugar pucker.

To get further insights into the function of the bulge conformation in the branching reaction the sugar pucker of A23 in both states was investigated. First, the D56 RNA construct was trapped in either one of the two bulge conformations by nucleotide mutations. The G6C and C21G mutations lead to a strong preference of 1B as in the competing 2B fold a C6–C20 and a G5–G21 mismatch will occur. The NMR data is fully in support of a switching towards 1B (Figure 4A, Supplementary Figure S10). Similarly, by the introduction of the G6C/C20G mutations a C6–C19 and a G7–G20 mismatch are introduced in 1B and 2B is now exclusively populated (Figure 4D, Supplementary Figure S10).

By using the ^13^C version of a H(N)HA-NMR experiment, (30,33) here termed H1′(C1′)H2′ experiment, the sugar puckers of ^13^C1′-labeled U12 and ^13^C1′-labelled A23 in the 1B and 2B mutant were addressed. The U12 residue served as an internal standard as in the cUUCGg tetraloop the second loop uridine is known to populate the C2′-endo conformation (34,35). The nmrpipe *nlinLS* peak fitting procedure was used to determine the peak intensities of the diagonal and cross peaks and the ^3^*J*_H1’H2’_ coupling constants of A23 and U12 in the 1B and 2B fold were obtained (29).

First, in 1B for U12 a scalar coupling constant of 8.8 Hz was found in-line with a high population of the C2′-endo sugar pucker, for A23–1B a coupling constant of 4.6 Hz was determined. Assuming a simplified model with a purely south/north sugar pucker equilibrium this would correspond to 46% C2′-endo sugar pucker of A23 in the 1B fold (Figure 4B and C) (36,37). In stark contrast, for the 2B G6C/C20G mutant no cross-peak was observed in the H1′(C1′)H2′ experiment for A23–2B and thus the ^3^*J*_H1’H2’_ coupling constant was not determinable (Figure 4D–F). This confirms an almost exclusive C3′-endo conformation of A23 in the 2nt bulge conformation. For U12, however, a cross-peak was found and a coupling constant of ca. 9 Hz was again confirmed.

Our data supports a functional sugar pucker switching between 1B and 2B. In fold 1B the A23 C2′-endo sugar pucker is the internal transesterification active conformation allowing step one—the branching reaction—in group II intron catalysis. This is in perfect agreement with a recent NMR and computational work, which showed that the ribose south conformation has a lower activation energy in an RNA cleavage step via the internal transesterification reaction (38). Once step one is accomplished the lariat structure is put in a resting state by switching into the north C3′-endo conformation and the group II intron can prepare for the exon ligation step.

### Various D6 constructs display structural heterogeneity on various time scales

Six group II intron domain 6 constructs from various organisms were tested for the occurrence of the secondary structure equilibrium. Thereby, we wanted to rule out that the secondary structure equilibrium observed in the *Oceanobacillus iheyenis/Azotobacter vinelandii* D6 chimera is an artefact originating from this very special sequence context. In detail, we investigated the folding behaviour of the *Oceanobacillus iheyensis* group II intron truncated domain 6, optimized for the X-ray structure determination by Pyle and co-workers, and compared it to the wildtype sequence (12). For this truncated 20nt D6 RNA with an 8–^13^C-labelled branch site adenosine we found a single ^1^H–^13^C correlation (Supplementary Figure S11A and B) and also the imino proton part of the ^1^H spectrum indicated a single fold. Strikingly, in the wildtype 35nt *OcI* D6 sequence we found again two H-C correlations for the branch site adenosine with an 8–^13^C-label and also low intensity imino proton signals pointed towards an additional fold. Integration of the HMQC correlation peaks gave a fold ratio of 7/3 similar to the fold distribution we found for the Oc19 D56 RNA. Using the *MC-sym* secondary structure prediction algorithm for the truncated D6 sequence a single fold was suggested, whereas for the 35nt wt construct two main folds with similar free energies were predicted in line with our experimental data (Supplementary Figure S11C and D) (39). We then incorporated the branch-site 8–^13^C-A label into four other D6 RNAs belonging to various classes, like the group IIC class and the bacterial group B and E class. For all D6 RNAs we observed structural heterogeneity either on the slow chemical shift time scale or as for the *Gracilibacillus* group IIC D6 on the intermediate exchange regime (Supplementary Figure S12). Our results suggest that the structural heterogeneity of the branch site adenosine in D6 is preserved in various group II intron classes. This points toward true functional dynamics, which is important for the catalytic mechanism of the ribozyme to switch between sugar puckers and conformations between the branching and exon ligation step, respectively.

## DISCUSSION

**Figure 5.**
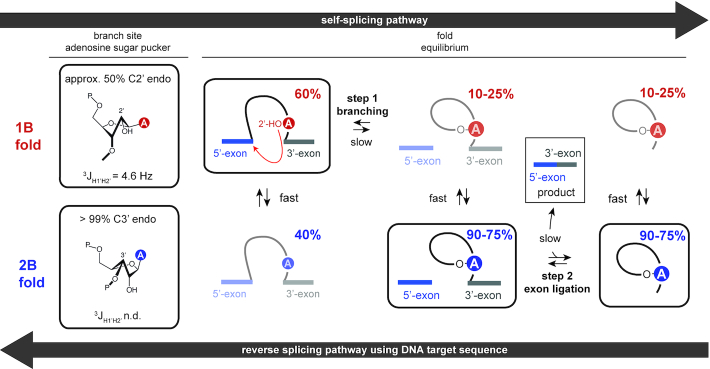
A linked secondary structure and sugar pucker equilibrium for the selection of catalytically relevant states. The sugar pucker equilibrium of the branch site adenosine and the secondary structure distributions during the two steps of group II intron catalysis are given. Inactive folds for the forward self-splicing pathway are shown transparent. These states are the active states in the reverse splicing pathway. Our data strongly supports a conformational selection mechanism in group II intron catalysis to guarantee the availability of reaction competent states for either the forward or backward reaction pathway.

Here, by characterizing secondary structure dynamics and a functional sugar pucker switching of the group II intron D6, a novel aspect in ribozyme catalysis was identified. Our data revealed the co-existence of two secondary structures with either a 1nt or a 2nt bulge in the 61nt *Oc19* D56 RNA. This conformational heterogeneity of D6 leads to a sugar pucker change of the branch site adenosine from the C2-’ to C3′-endo state, thereby modulating the transesterification activity. The findings suggest that the group II intron ribozyme utilizes a conformational selection mechanism to vary the population of reaction competent and incompetent states to orchestrate the various steps—branching and exon ligation—of group II intron catalysis. The secondary structure equilibrium is further proposed to be a key aspect for the reversibility in group II intron splicing. The findings of our work are summarized in a revised schematic representation of the branching pathway integrating the secondary structure and sugar pucker equilibrium in the splicing process (Figure 5).

Using solution NMR spectroscopy, we were able to unambiguously determine the slow exchange process of the two D6 secondary structures. Further, residue- and atom-specific ^15^N stable isotope labeling of the D56 RNA allowed to detect fold-specific Watson–Crick base pairs of both states, thus confirming the two secondary structure predictions.

The application of ^13^C longitudinal exchange NMR experiments revealed a hierarchical kinetic network with the fastest exchange rates observed at the bulge sites and a steady decrease of the folding rates towards the tetraloop. The kinetic data shed light on the forward folding pathway with an initial base pairing rearrangement process at the 1nt-bulge site of 1B, followed by a sequential base pairing register change in the G-C rich central tract culminating in fold 2B. The backward pathway again starts with the base pair switching in the labile 2nt-bulge sequence part, followed by the G-C base pair rearrangement in the central tract and closed by the formation of the symmetrical C-C bulge of 1B.

We then addressed the question how the secondary structure equilibrium of D6 can be modulated by external cues. A very strong equilibrium shift effect could be observed upon the addition of magnesium (II) ions. The added divalent cations lead to a preference of 2B, thus preparing the group II intron for the second exon ligation step. Magnesium (II) ions are essential co-factors for this step as catalytically relevant Mg^2+^ ions are found in D5—the catalytic centre of the second step (4). This suggests that by an increase of the magnesium ion concentration the group II intron switches its activity towards the exon ligation step. By establishing a synthetic access to a branched RNA mimic the influence of additional nucleotides attached to the branch site adenosine 2′-oxygen on the secondary structure equilibrium was probed. The branching reaction product favors the 2nt-bulge, which gets even more pronounced in the presence of magnesium (II) ions where the 2B fold is populated at 90%. This equilibrium shift is again in perfect accordance with the assumption that 1B is the active conformation in the first step, followed by switching to 2B by branching to facilitate step two. We also determined the sugar pucker of the branch site adenosine in both the 1B and 2B state. We found a switching from approximately 50% C2′-endo in 1B to an exclusive C3′-endo conformation in 2B. In 1B the adenosine C2′-endo sugar pucker population is the active conformation allowing the branching reaction, as this ribose south state has a lower activation energy in an RNA cleavage step via the internal transesterification reaction (38). Then, the branched RNA structure is put in a resting state by switching into the north conformation and the exon ligation can occur. Our data gives a molecular level explanation for the results of earlier conducted splicing assays on mutants favoring one or the other fold/sugar pucker and thus activating either the branching or exon ligation step (16).

The structural heterogeneity could be recapitulated in five other D6 RNA constructs from various classes suggesting that we indeed observe functional secondary structure and sugar pucker dynamics in D6 with an important role in catalysis.

Taken together, our findings strongly suggest that conformational dynamics is a crucial aspect in group II intron self-splicing. So far, the conformational selection concept was mainly discussed in context of biomolecular recognition and in protein catalysis but not as a key aspect in ribozyme activity (40–49). The dynamic nature of D6 leads to a tuneable abundance of the reaction competent state of the branch site adenosine by re-shuffling the equilibrium populations by specific triggers. Thereby, the ribozyme can move along the reaction coordinate in both directions, thus giving a rational for the reversibility in group II intron splicing. Although we were able to identify some of the triggers to switch the fold/sugar pucker equilibrium within D6, such as magnesium (II) ions or lariat formation, it is very tempting to speculate that another very important group II intron cofactor—the group II intron protein component—could dynamically modulate the secondary structure and the sugar pucker equilibrium in D6 as it was recently shown that the maturase protein is important for the active site substrate exchange (15,50,51). Furthermore, transient RNA-RNA contacts such as the π–π’ or the η–η’ interaction between D2 and D6 could regulate the populations of the secondary structure equilibrium to switch on and off functional states in splicing (10).

## Supplementary Material

gkz965_Supplemental_File

## Notes

Present address: Michael Andreas Juen, Roche Diagnostics GmbH, Nonnenwald 2, 82377 Penzberg, Germany.
