# Supplementary material for: Branch site bulge conformations in domain 6 determine functional sugar puckers in group II intron splicing
Source: Nucleic Acids Res. 2019 Oct 29;47(21):11430–40. doi: 10.1093/nar/gkz965 (PMC6868427; doi:10.1093/nar/gkz965)
Supplement: gkz965_Supplemental_File [file gkz965_supplemental_file.pdf]

SUPPLEMENTARY INFORMATION

Branch site bulge conformations in domain 6 determine functional sugar puckers in group II intron splicing

Raphael Plangger[1], Michael Andreas Juen[1,+], Thomas Philipp Hoernes[2], Felix Nußbaumer[1], Johannes Kremser[1], Elisabeth Strebitzer[1], David Klingler[1], Kevin Erharter[1], Martin Tollinger[1], Matthias David Erlacher[2] and Christoph Kreutz[1,*]

[1] Institute of Organic Chemistry and Center for Molecular Biosciences Innsbruck (CMBI), University of Innsbruck, Innrain 80/82, 6020 Innsbruck, Austria.

2 Division of Genomics and RNomics, Biocenter, Medical University of Innsbruck, Innrain 80/82, 6020 Innsbruck, Austria.

Corresponding author: Christoph Kreutz (christoph.kreutz@uibk.ac.at)

The authors wish it to be known that, in their opinion, the first two authors should be regarded as joint First Authors.

[+] Present Address: Michael Andreas Juen, Roche Diagnostics GmbH, Nonnenwald 2, 82377 Penzberg, Germany.

SUPPLEMENTARY RESULTS

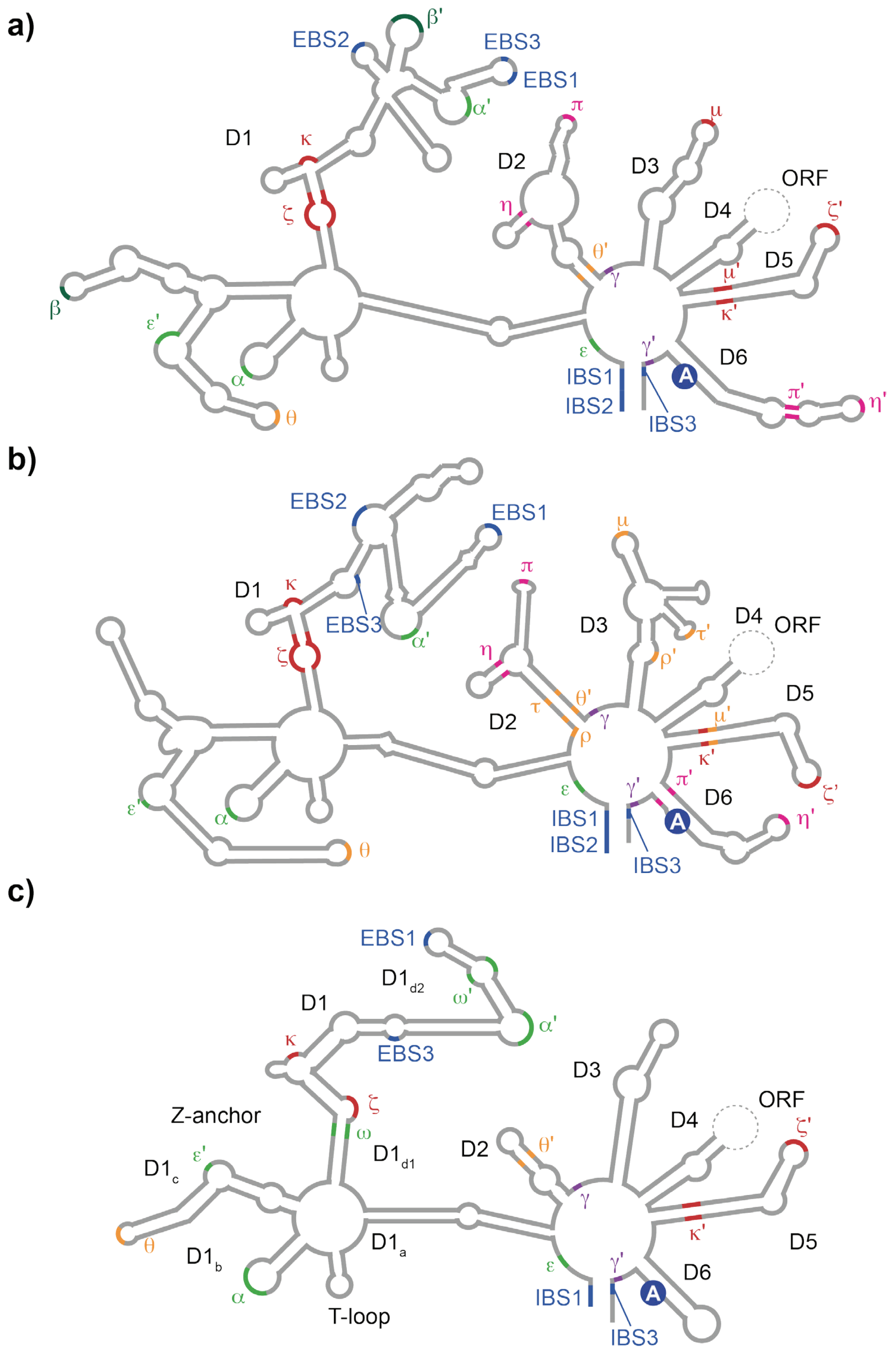

Supplementary Figure 1. Domain organization for group II introns from class IIA, class IIB and class IIC. **a)** Schematic representation of group II intron subclass IIA. **b)** Schematic representation of group II intron subclass IIB. **c)** Schematic representation of group II intron subclass IIC. Domains are annotated with D1 to D6, greek letters indicate long range interactions, EBS exon binding sites, IBS intron binding sites, ORF open reading frame, branch site A in domain 6 is highlighted.

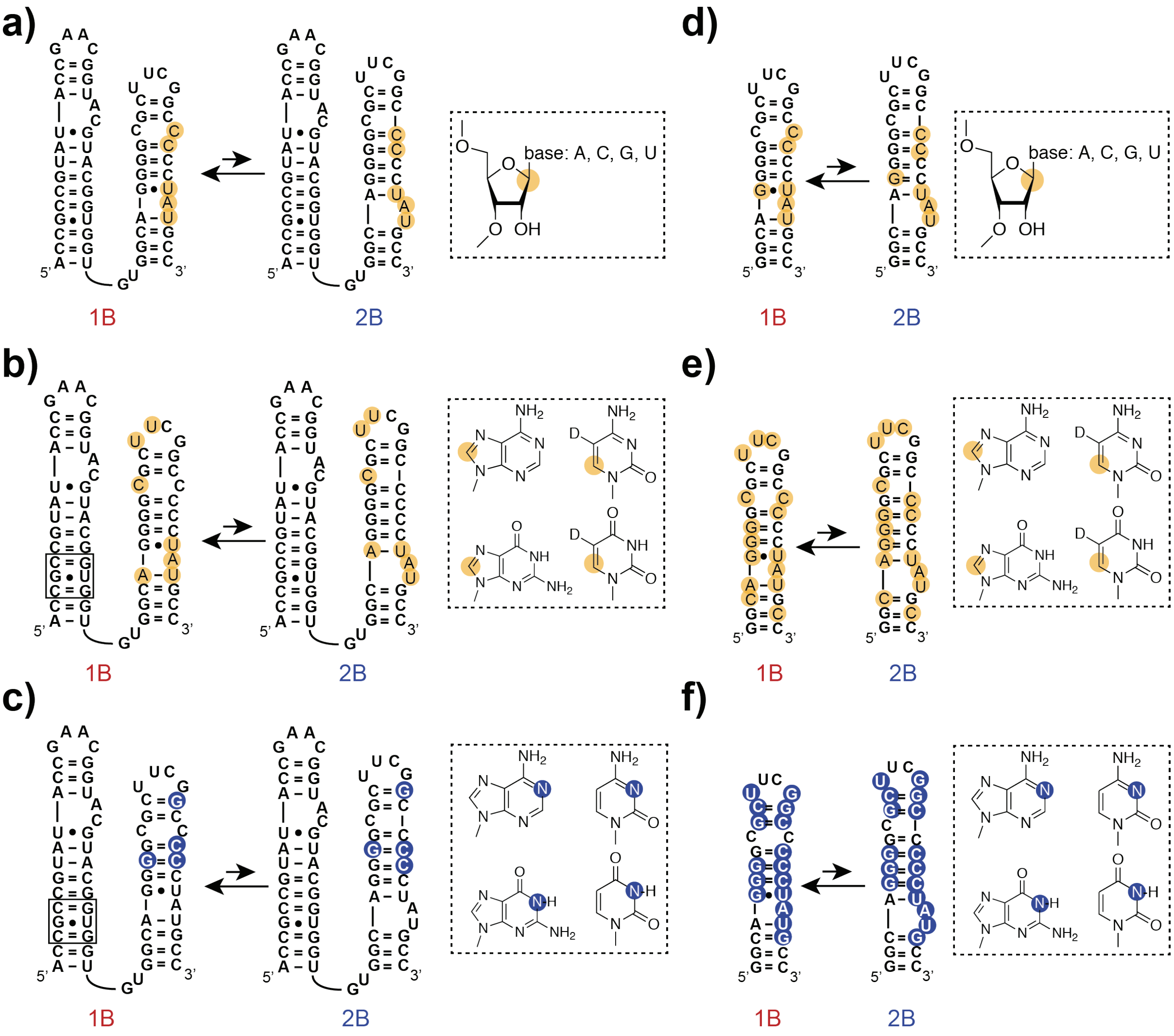

Supplementary Figure 2. Site-specific SI labeled RNAs used in this work. **a)** Sugar ^{13}C1'-labels in the 61nt D56 RNA. **b)** Aromatic ^{13}C6-pyrimdine and ^{13}C8-purine-labels in the 61nt D56 RNA. **c)** ^{15}N3-pyrimdine and ^{15}N1-purine-labels in the 61nt D56 RNA. **d)** Sugar ^{13}C1'-labels in the 27nt D6 RNA. **e)** Aromatic ^{13}C6-pyrimdine and ^{13}C8-purine-labels in the 27nt D6 RNA. **f)** ^{15}N3-pyrimdine and ^{15}N1-purine-labels in the 27nt D6 RNA.

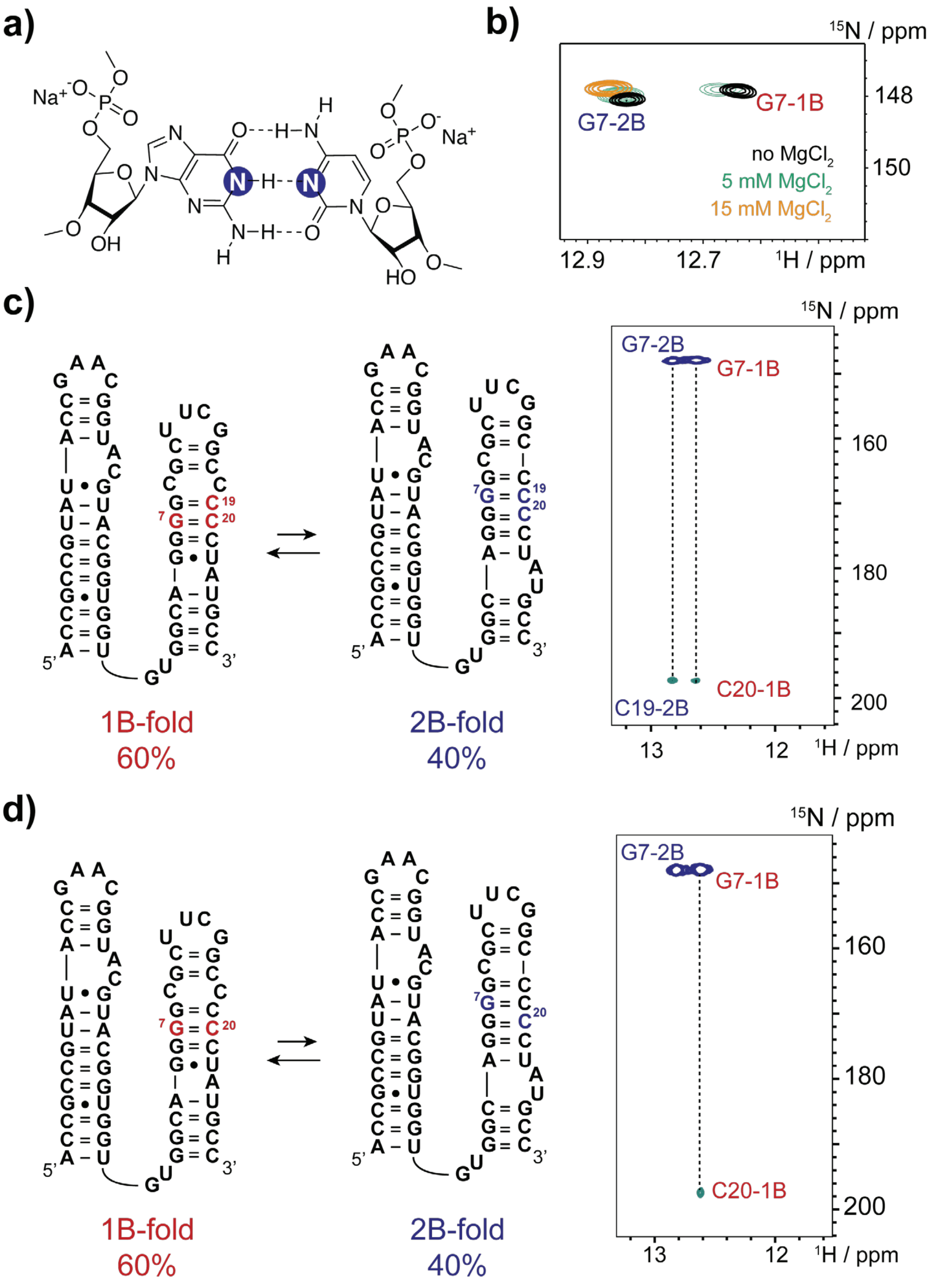

Supplementary Figure 3. Detection of the fold-specific Watson Crick base pairs G7-1B//C20-1B and G7-2B//C19-2B. **a)** ^{15}N-labeling pattern for the detection of fold specific G-C Watson-Crick base pairs. Blue dot = ^{15}N. **b)** ^{1}H-^{15}N-SOFAST HMQC spectrum of ^{15}N1-G7 labeled 61nt D56 RNA. Fold 2B gets favored upon the addition of magnesium (II) chloride. **c)** HNN-COSY experiment of ^{15}N1-G7, ^{15}N3-C20 and ^{15}N3-C19 labeled D56 RNA unambiguously confirming the coexistence of the G7-C20 base pair in fold 1B and G7-C19 base pair in 2B. **d)** HNN-COSY experiment of ^{15}N1-G7 and ^{15}N3-C20 labeled D56 RNA for resonance assignment purposes. Only the G20-C19 base pair of fold 1B gives a correlation between the donor and acceptor nitrogen and the G7 H1 imino proton.

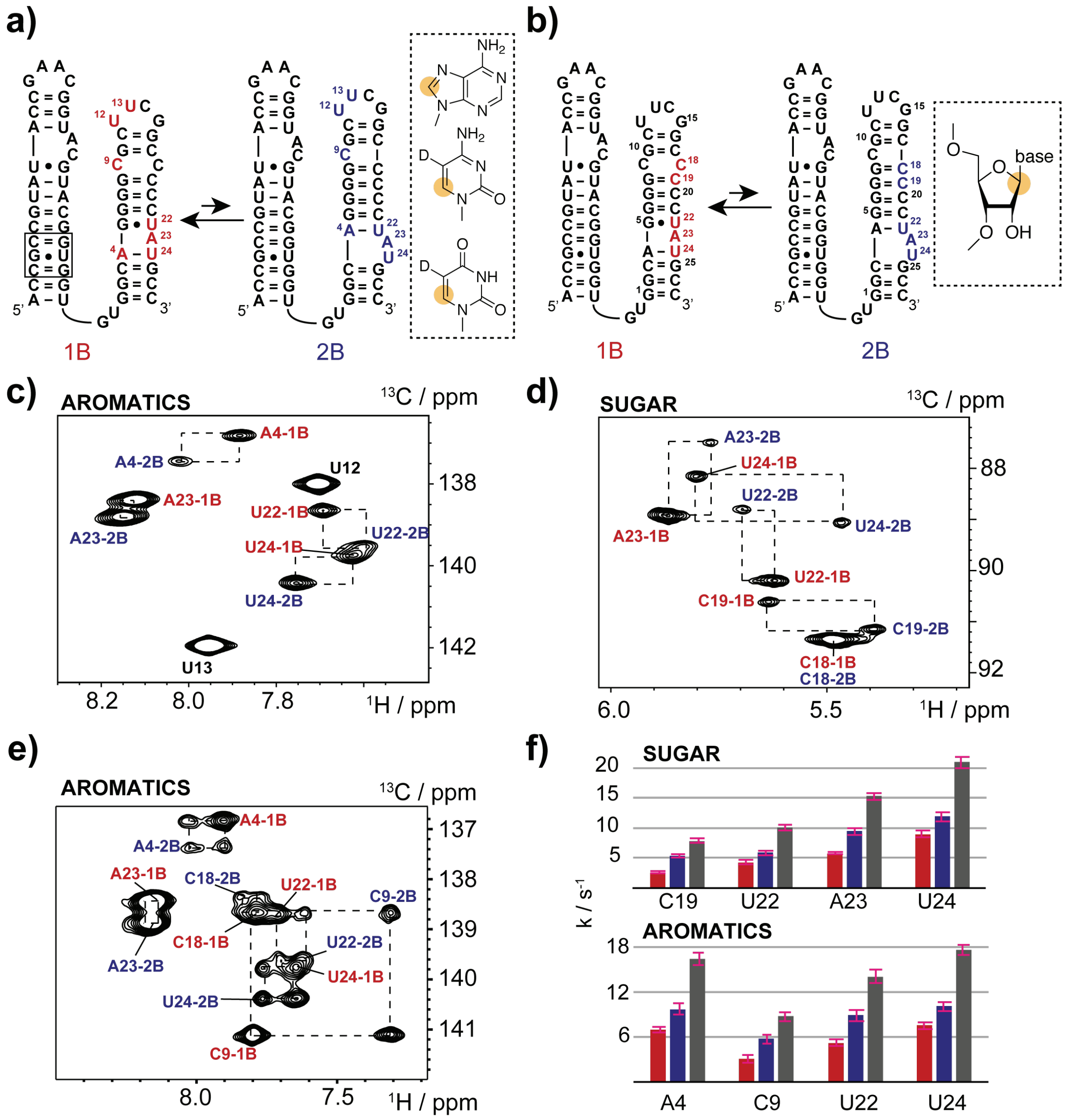

Supplementary Figure 4. Fold heterogeneity in the D56 RNA analyzed by solution NMR spectroscopy. **a)** Secondary structure representation of folds 1B and 2B with ^{13}C8/^{13}C6-nucleobase labeled residues highlighted in red (1B) and blue (2B), respectively. The inset shows the labeled aromatic carbons (orange dot). **b)** Secondary structure representation of folds 1B and 2B with ^{13}C1'-sugar labeled residues highlighted in red (1B) and blue (2B), respectively. The inset shows the labeled C1'-carbon (orange dot). **c)** A ^{1}H-^{13}C HMQC spectrum of the ^{13}C6-U12, U13, U24 and ^{13}C8-A4 and A23 labeled D56 RNA construct. The individual resonances were assigned using residue specific labeling by chemical solid phase synthesis of fold specific mutants (G6C/C21G fold 1B and G6C/C20G fold 2B). **d)** A ^{1}H-^{13}C HMQC spectrum of the ^{13}C1'-C18, C19, U22, A23 and U24 labeled D56 RNA construct. The individual resonances were assigned as in c. **e)** A ^{13}C-ZZ exchange NMR spectrum of an

optimized nucleobase labeled D56 RNA construct to minimize resonance overlap (^{13}C6-C9, C18, U22 and U24, ^{13}C8-A4 and A23) at a mixing time of 80 ms. **f)** Bar plots of exchange rate constants in s^{-1} determined from aromatic and sugar ^{13}C ZZ exchange NMR experiments. Red bar forward rate constant k_f, blue bar backward rate constant k_b and grey bar exchange rate $k_{ex} = k_f + k_b$.

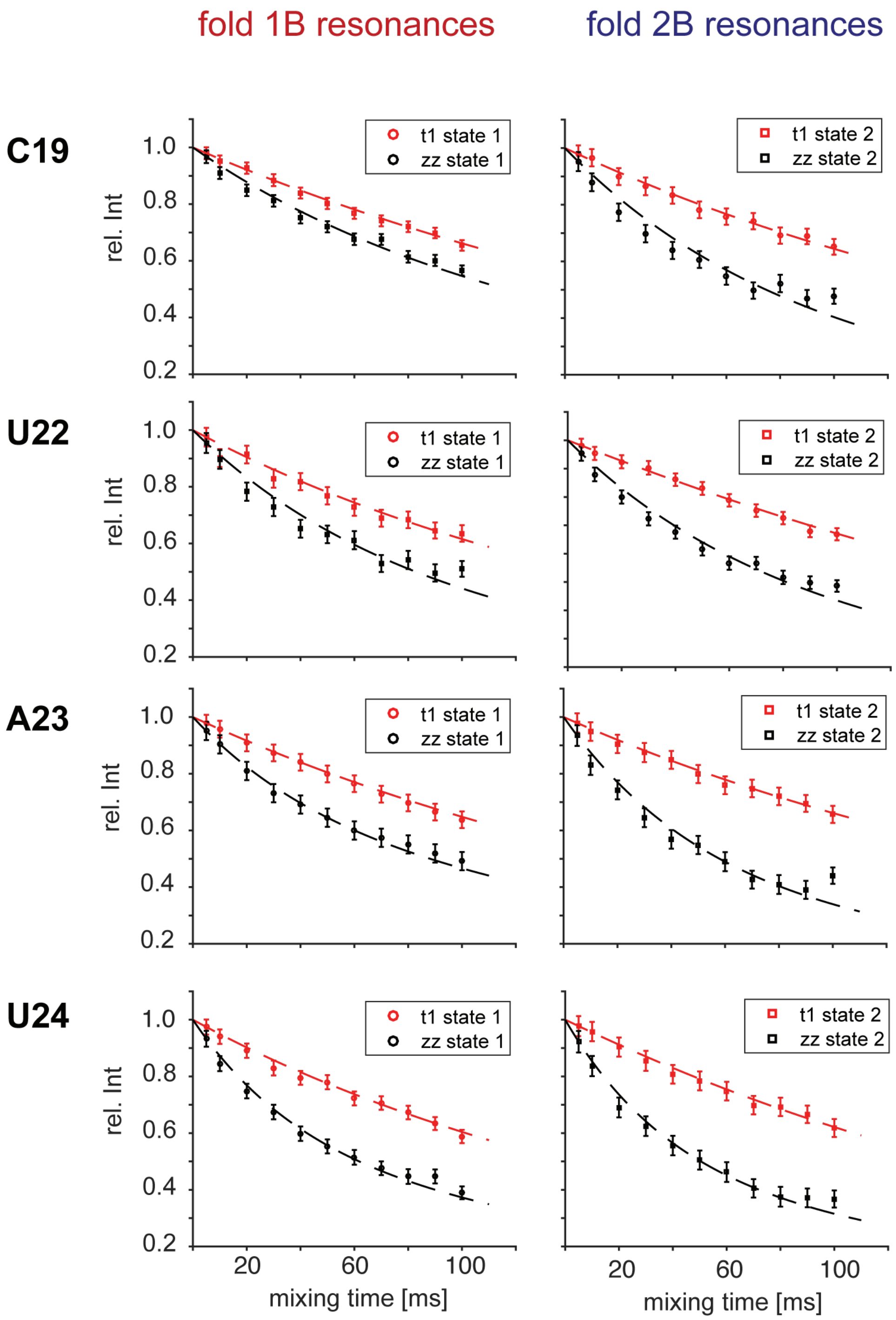

Supplementary Figure 5. T_1 and ZZ relative intensity decays of ^{13}C1'-labeled D56 RNA used for the extraction of exchange rates and longitudinal relaxation rates.

Supplementary Table 1. Summary of kinetic data (forward and backward rate constants) from ^{13}C ZZ exchange NMR experiments for sugar $^{13}C1'$ and $^{13}C6$ pyrimidine and $^{13}C8$ purine labeled D56 and D6 only RNAs.

RNA	residue	k_{ex} / s^{-1} [a]	$k_{1B\text{-}2B}$ / s^{-1} [b]	$k_{2B\text{-}1B}$ / s^{-1} [c]	p_{1B} / % [d]	p_{2B} / % [d]
**D56 61nt**	C19 C1'	7.88 ± 0.39	2.55 ± 0.21	5.33 ± 0.33	68 ± 2	32 ± 2
	U22 C1'	10.11 ± 0.47	4.28 ± 0.37	5.83 ± 0.30	58 ± 2	42 ± 2
	A23 C1'	15.24 ± 0.57	5.81 ± 0.26	9.42 ± 0.50	62 ± 2	38 ± 2
	U24 C1'	20.89 ± 0.91	9.00 ± 0.54	11.89 ± 0.73	57 ± 1	43 ± 1
**D56 61nt**	A4 C8	16.45 ± 0.86	6.98 ± 0.44	9.47 ± 0.74	58 ± 2	42 ± 2
	C9 C6	8.74 ± 0.59	3.02 ± 0.53	5.72 ± 0.64	53 ± 4	47 ± 4
	U22 C6	14.10 ± 0.88	5.21 ± 0.47	8.88 ± 0.74	63 ± 2	37 ± 2
	U24 C6	17.62 ± 0.72	7.56 ± 0.46	10.06 ± 0.56	57 ± 2	43 ± 2
**D6 27nt**	A4 C8	19.84 ± 1.29	7.85 ± 0.76	12.00 ± 1.01	60 ± 2	40 ± 2
	C9 C6	9.06 ± 0.59	3.84 ± 0.39	5.22 ± 0.44	68 ± 3	32 ± 3
	C18 C6	4.45 ± 0.46	1.66 ± 0.29	2.79 ± 0.35	63 ± 5	37 ± 5
	A23 C8	12.72 ± 1.35	4.09 ± 0.63	8.62 ± 1.19	58 ± 3	42 ± 3
	U24 C6	16.09 ± 0.87	6.80 ± 0.54	9.29 ± 0.68	68 ± 2	32 ± 2

[a] $k_{ex} = k_{1B\text{-}2B} + k_{2B\text{-}1B}$ in s^{-1}; [b] forward rate constant $k_{1B\text{-}2B}$ in s^{-1}; [c] backward rate constant $k_{2B\text{-}1B}$ in s^{-1}; [d] populations of fold 1B (p_{1B}) and 2B (p_{2B}).

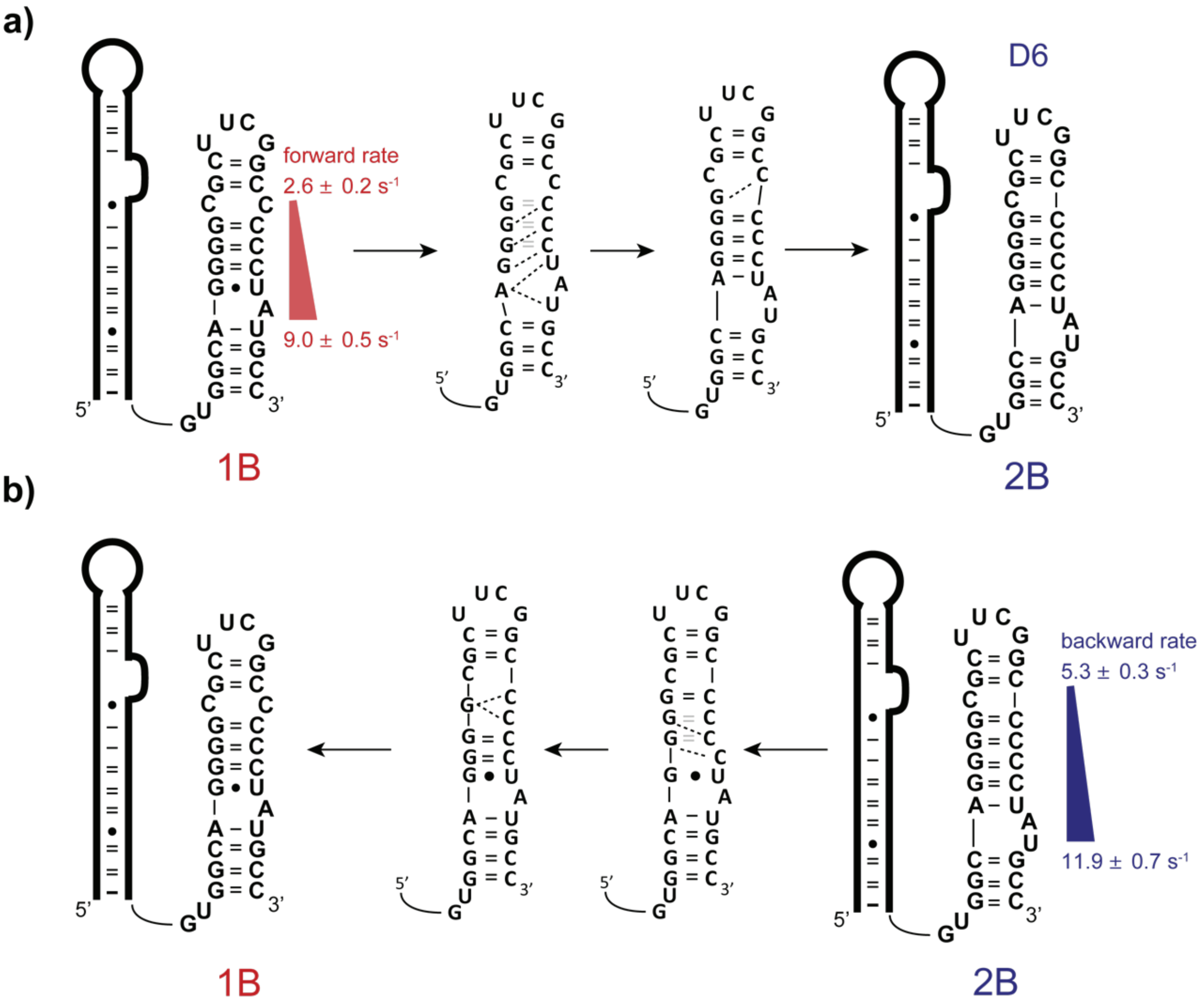

Supplementary Figure 6. Forward and backward folding pathway of D56 RNA. **a)** Forward pathway from fold 1B to 2B. **b)** Backward pathway from fold 2B to 1B. For details refer to the main text.

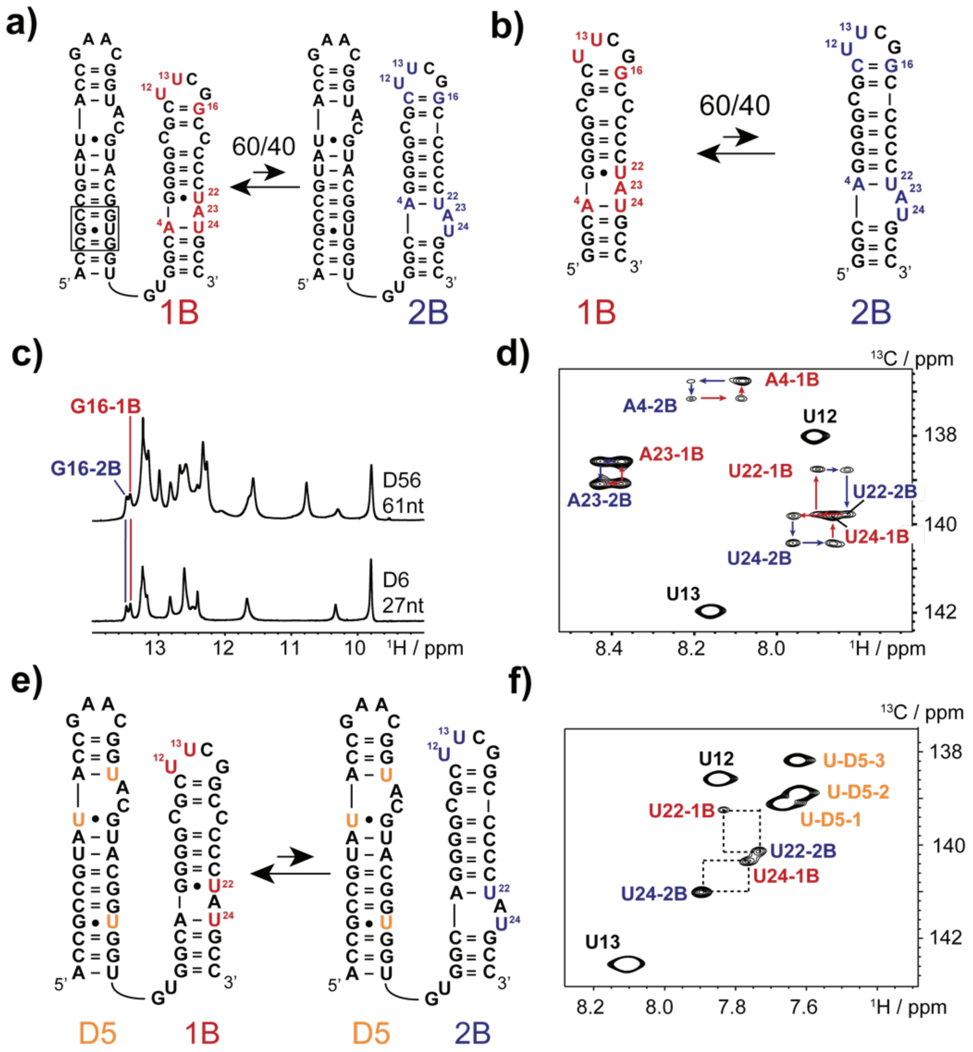

Supplementary Figure 7. The 1B and 2B fold heterogeneity is existent in both the 61nt D56 RNA and in a 27nt D6 only RNA construct. **a)** Secondary structure representation of 61nt D56 RNA with fold 1B and 2B in equilibrium. From peak integration and kinetic data an equilibrium position of 60/40 was determined. **b)** Secondary structure representation of 27nt D6 RNA with fold 1B and 2B in equilibrium. From peak integration and kinetic data an equilibrium position of 60/40 was determined, which is identical to the behavior of the D56 RNA. **c)** Imino proton spectrum comparison of D56 and D6 RNA. The D6 imino proton resonances can be also found in the D56 RNA indicating a highly modular behavior of the D56 RNA. **d)** A ^{13}C-ZZ exchange NMR spectrum of a ^{13}C8-A4 and A23 and a ^{13}C6-U12, U13, U22 and U24 D6 RNA construct at a mixing time of 125 ms. **e)** Secondary structure representation of 61nt D56 RNA with ^{13}C6-U labels highlighted in orange (D5), red (D6, 1B fold) and blue (D6, fold 2B). **f)** A ^{1}H-^{13}C HMQC spectrum of a ^{13}C6-U labeled D56 RNA. The U22 and U24 ^{1}H-^{13}C-resonances in D6 show slow exchange on the chemical shift time scale, whereas the D5 U and the D6 U12 and U13 residues give rise to a single C/H signal localizing the 2° structure and sugar pucker equilibrium in D6.

Supplementary Table 2. Magnesium (II) ion and EDTA dependent equilibrium populations of 1B and 2B from the integration of the A4 $^{1}H8$-$^{13}C8$-correlation peaks in the aromatic HMQC spectra.

Equivalents $MgCl_2$/EDTA	A4 $^{1}H8$-$^{13}C8$-peak integral **1B** / A.U.	A4 $^{1}H8$-$^{13}C8$-peak integral **2B** / A.U.	population **1B** / %	population **2B** / %
0/0	5785300	388605040	60	40
2/0	2473500	1822500	52	48
10/0	9067000	13016000	40	60
20/0	6303200	14346000	30	70
20/24	22120000	7207800	75	25

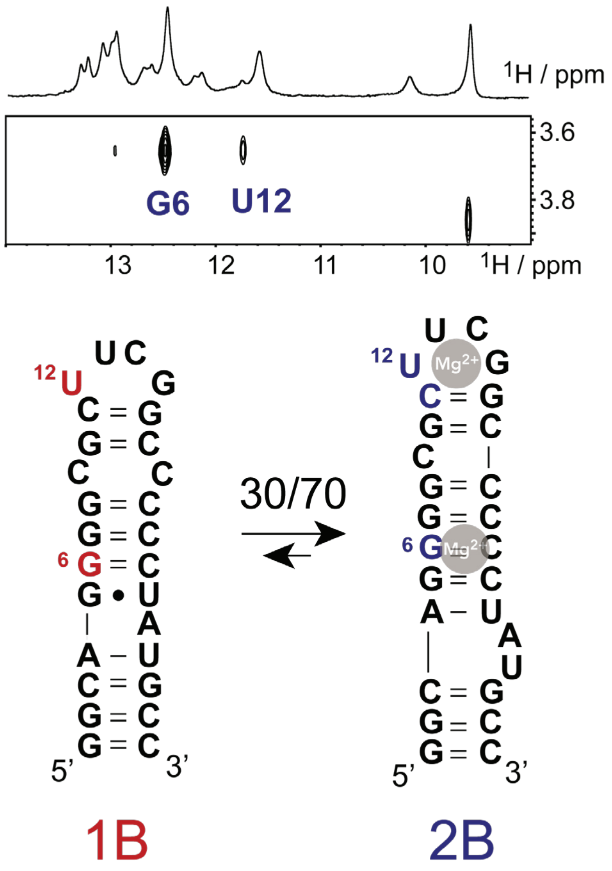

Supplementary Figure 8. Imino NOESY experiment in the presence of 10 mM $MgCl_2$ and 2 mM $Co(NH_3)_6Cl_3$. A NOE between an imino proton and the ammine proton signal at ca. 3.65 ppm indicates a cation binding site. Two NOEs of the G6-H1-2B and U12 H3 could be unambiguously identified allowing to localize potential magnesium (II) binding sites in the 27nt D6 RNA.

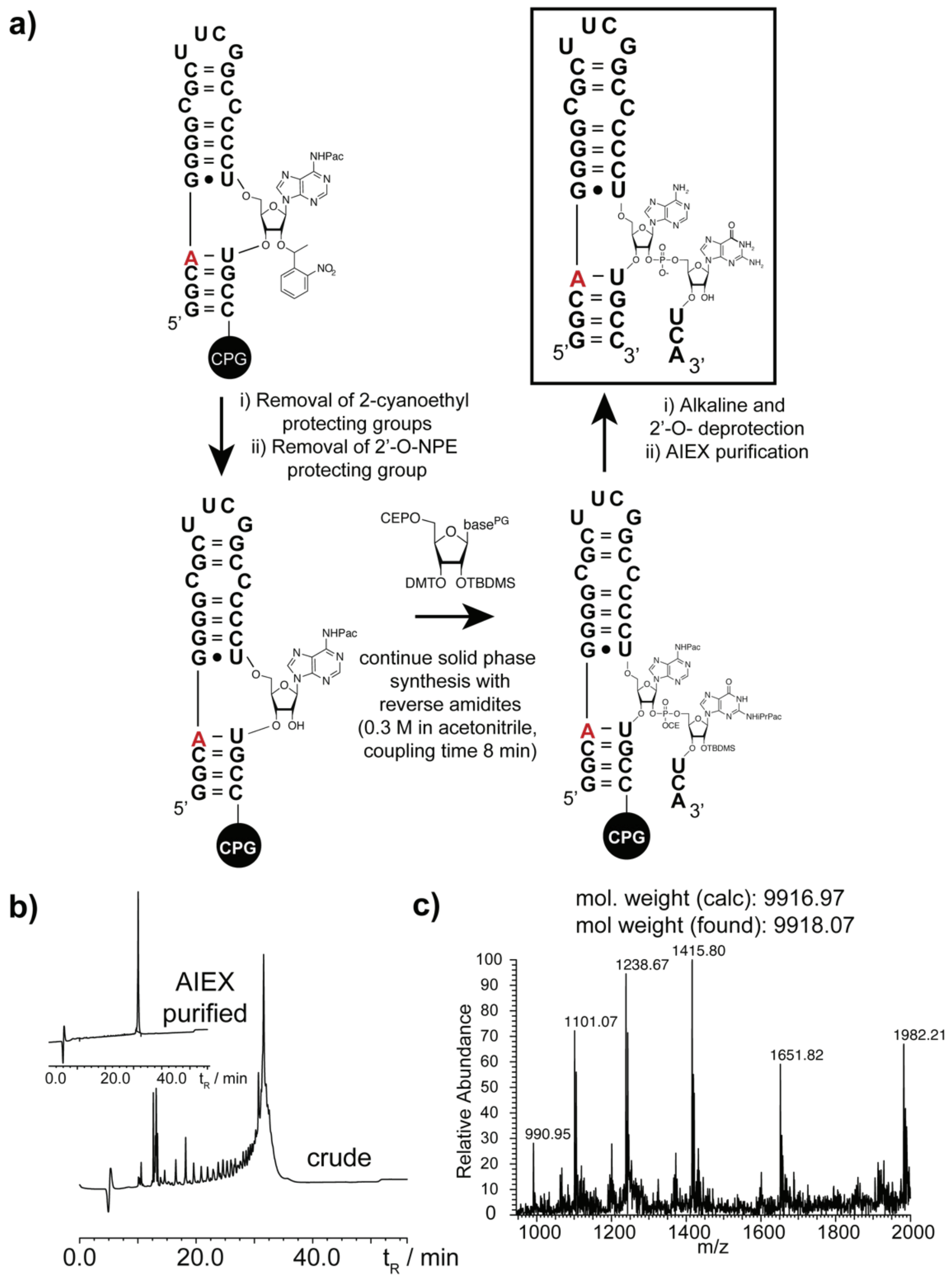

Supplementary Figure 9. Synthetic access to branched RNA mimic. **a)** The branch site adenosine carries a 2'-O-photolabile NPE protecting group. After removal of 2-cyanoethyl groups from the phosphate backbone, the NPE is removed by exposure to UV light (260 nm). Then, the branched RNA mimic is produced by using commercially available reverse phosphoramidites. After complete assembly the branched RNA is deprotected and purified according to the protocol described in the online methods section. The ^{13}C8-labeled adenosine A4 is highlighted in red. **b)** Anion-exchange chromatogram of crude branched RNA, and after purification by preparative anion exchange chromatography. **c)** ESI mass spectrum of purified branched D6 RNA.

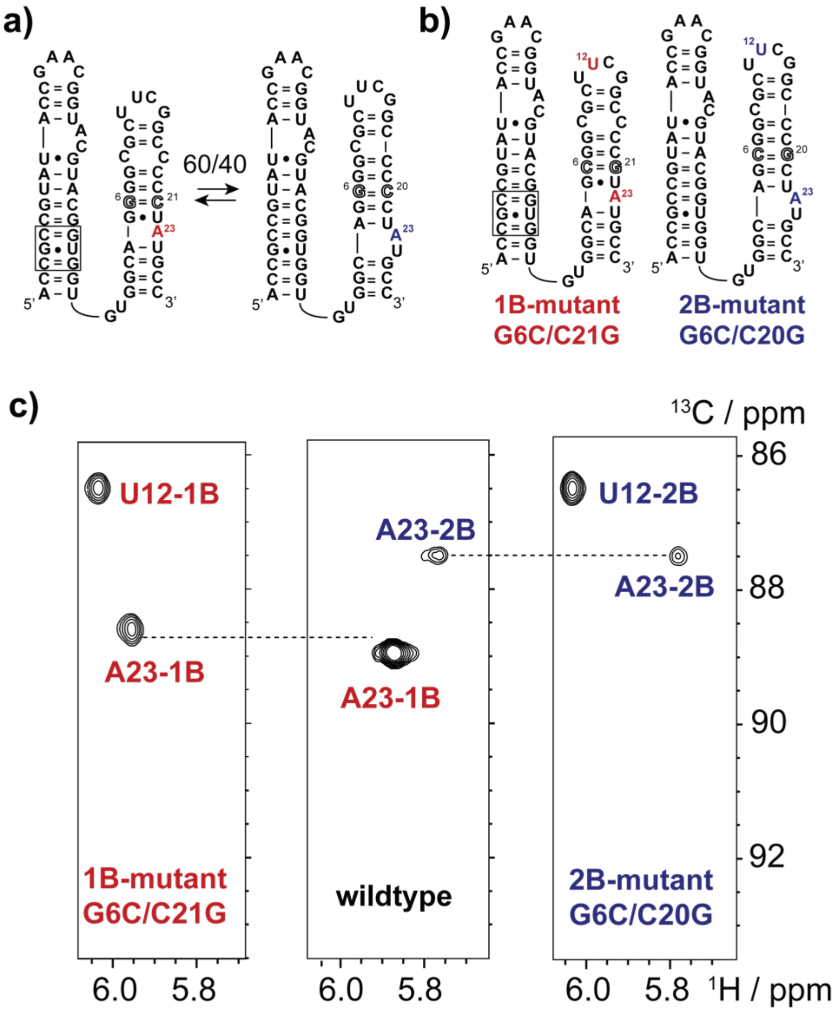

Supplementary Figure 10. Nucleotide mutations to trap the alternate branch site folds. **a)** Secondary structures of wildtype D56 RNA in fold 1B and 2B. The ^{13}C1'-labeled A23 is highlighted in red and blue, respectively. The mutation sites are shown in outlined letters. **b)** Secondary structures of fold 1B mutant G6C/C21G and fold 2B mutant with ^{13}C1'-labeled A23 and U12 highlighted in red and blue, respectively. The mutation sites are shown in outlined letters. **c)** ^{1}H-^{13}C-HMQC spectra confirming the trapping of the alternate branch site conformations by the nucleotide mutations.

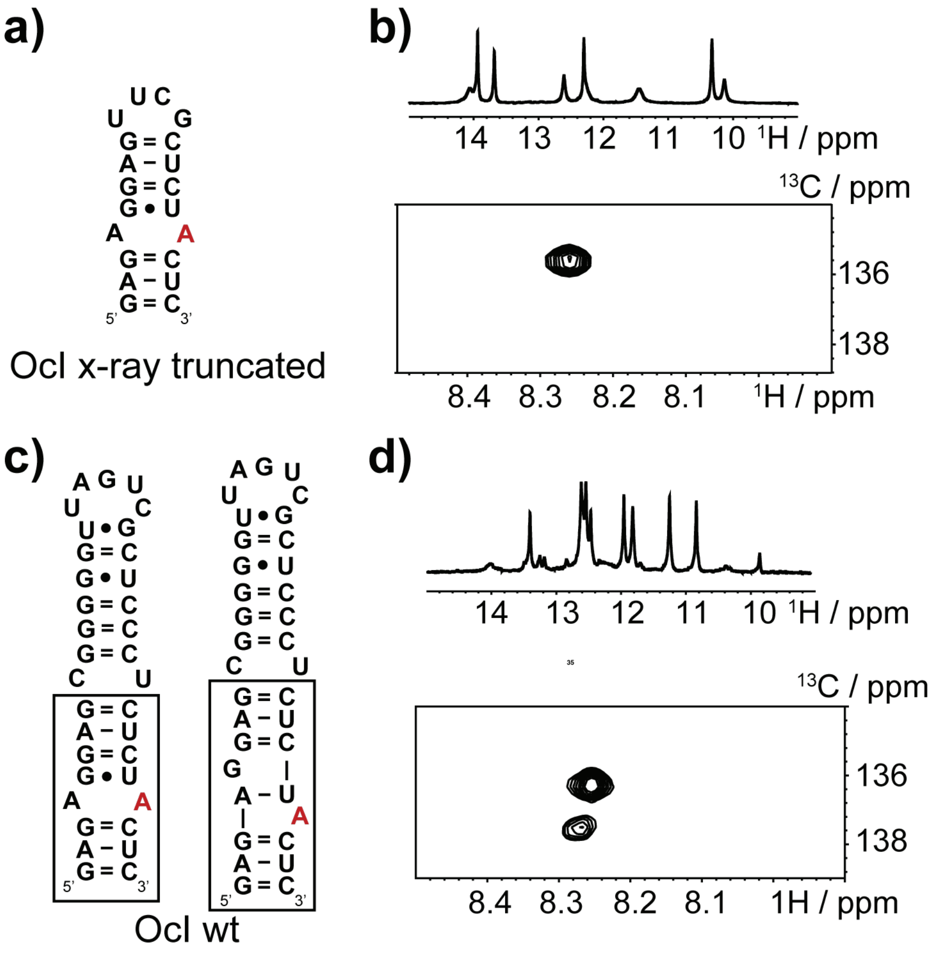

Supplementary Figure 11. Fold heterogeneity in the *Oceanobacillus iheyensis* group II intron truncated and the wildtype domain 6. **a)** Secondary structure representation of the truncated D6 RNA used for X-ray crystallography with the ^{13}C8-labeled branch site A highlighted in red. **b)** A ^{1}H-^{13}C-HMQC with a single peak for the branch site adenosine H/C resonance. **c)** Secondary structure representations of the wildtype *Oceanobacillus iheyensis* D6 RNA with the ^{13}C8-labeled branch site A highlighted in red. The box indicates the sequence part used for X-ray crystallography. **d)** A ^{1}H-^{13}C-HMQC with two peaks for the branch site adenosine H/C resonance indicative of fold heterogeneity on the slow chemical shift time scale.

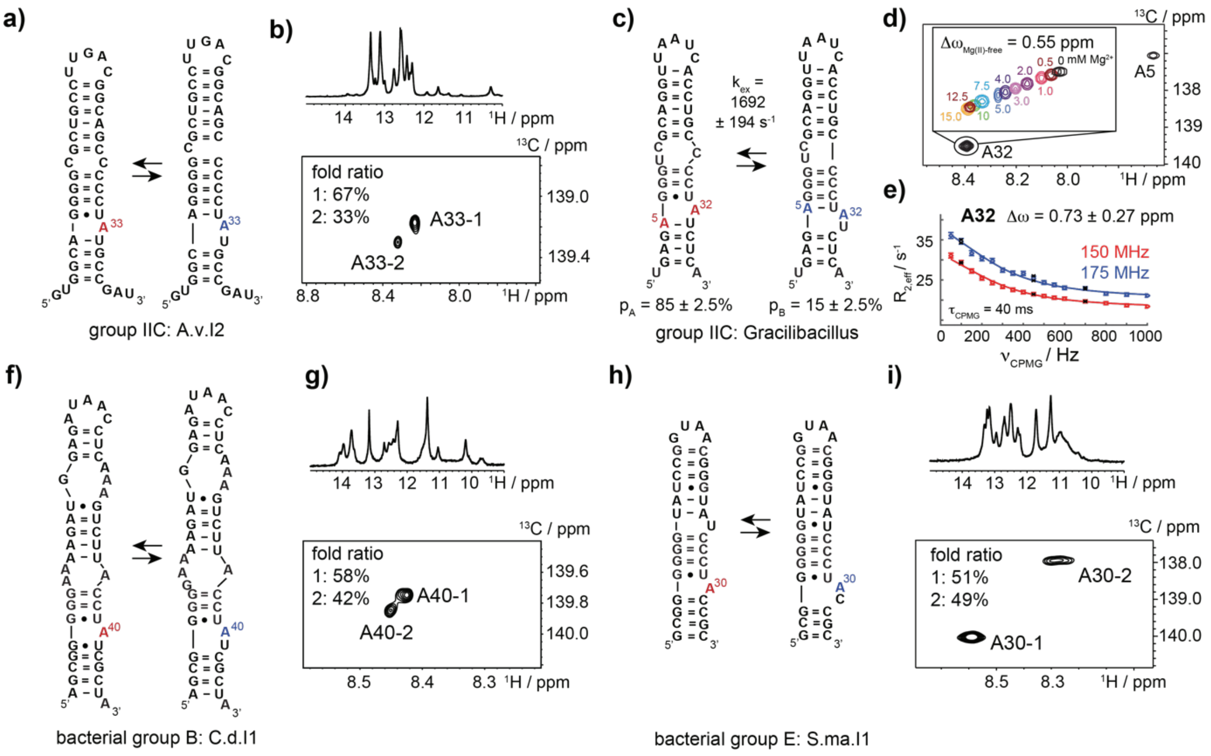

Supplementary Figure 12. Fold heterogeneity in various other group II intron subclasses. **a)** Secondary structures of 1B and 2B fold of *Azotobacter vinlandii* (A.v.I2) group II intron D6 from subclass IIC. **b)** Imino proton NMR spectrum and ^{1}H-^{13}C-HMQC spectrum of ^{13}C8-A33 labeled A.v.I2 D6 RNA pointing towards fold heterogeneity. **c)** Secondary structures of 1B and 2B fold of *Gracilibacillius* group II intron D6 from subclass IIC. **d)** ^{1}H-^{13}C-HMQC spectrum of ^{13}C8-A5 and ^{13}C8-A32 labeled *Gracilibacillus* D6 RNA. Addition of a magnesium (II) chloride leads to a strong shift of the A32 resonance. **e)** ^{13}C-CMPG relaxation dispersion indicating microsecond time scale dynamics of A32 and A5 (data not shown) in-line with the proposed fold heterogeneity. **f)** Secondary structures of 1B and 2B fold of *Clostridium difficile* (C.d.I1) group II intron D6 from bacterial group B. **g)** Imino proton NMR spectrum and ^{1}H-^{13}C-HMQC spectrum of ^{13}C8-A40 labeled C.d.I1 D6 RNA pointing towards fold heterogeneity. **h)** Secondary structures of 1B and 2B fold of *Serratia marcescens* (S.ma.I1) group II intron D6 from bacterial group E. **i)** Imino proton NMR spectrum and ^{1}H-^{13}C-HMQC spectrum of ^{13}C8-A30 labeled S.ma.I1 D6 RNA pointing towards fold heterogeneity.

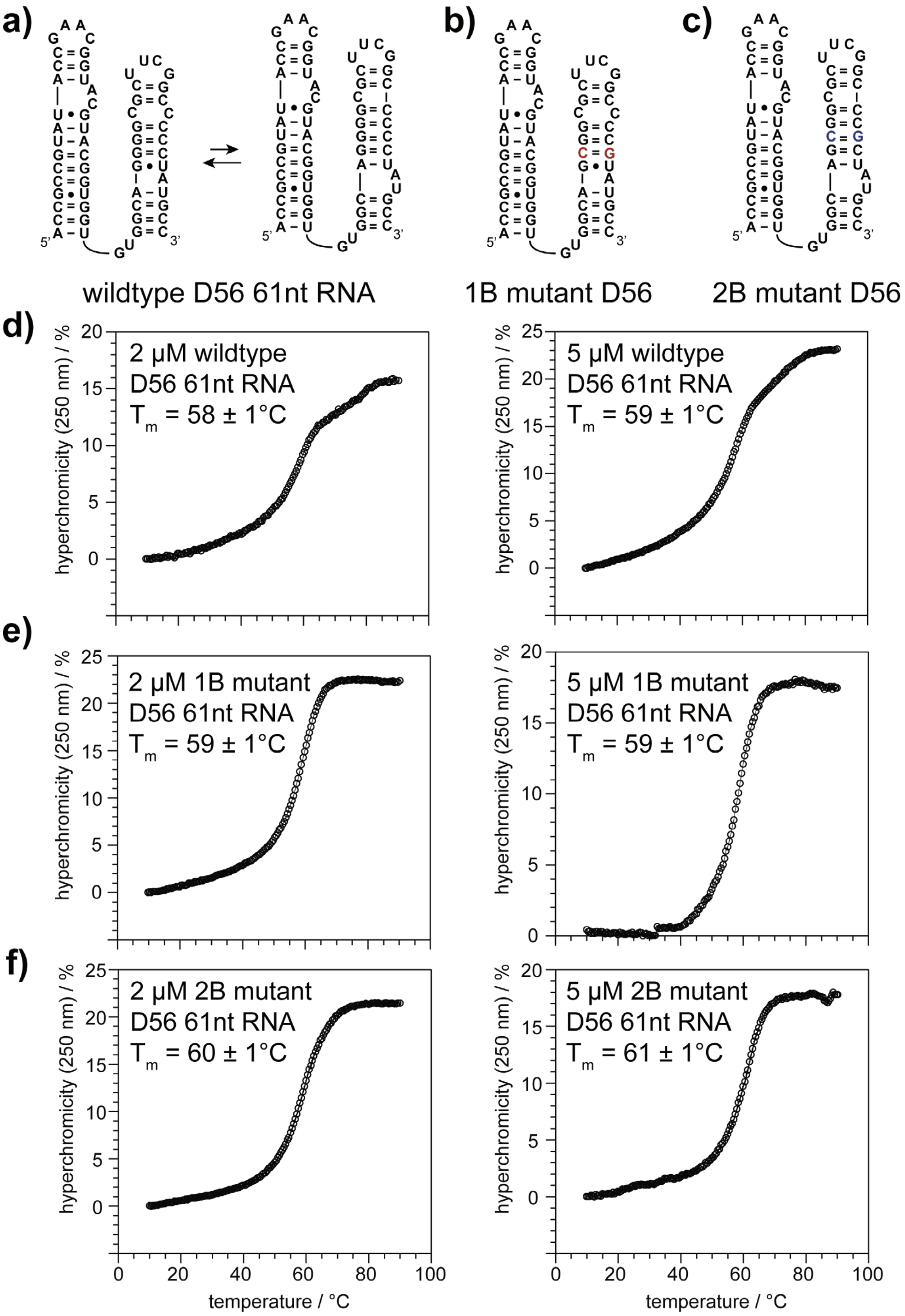

Supplementary Figure 13. UV melting curve analysis of the wildtype D56 RNA the 1B mutant D56 and the 2B D56 mutant. **a)** Secondary structures of wildtype D56 RNA. **b)** Secondary structures of 1B mutant D56 RNA. **c)** Secondary structures of 2B mutant D56 RNA. **d)** UV melting curves (250 nm, 2nd cooling curve) and at total RNA strand concentrations as indicated of wildtype D56 RNA. **e)** UV melting curves (250 nm, 2nd cooling curve) and at total RNA strand concentrations as indicated of 1B mutant D56 RNA. **f)** UV melting curves (250 nm, 2nd cooling curve) and at total RNA strand concentrations as indicated of 2B mutant D56 RNA.

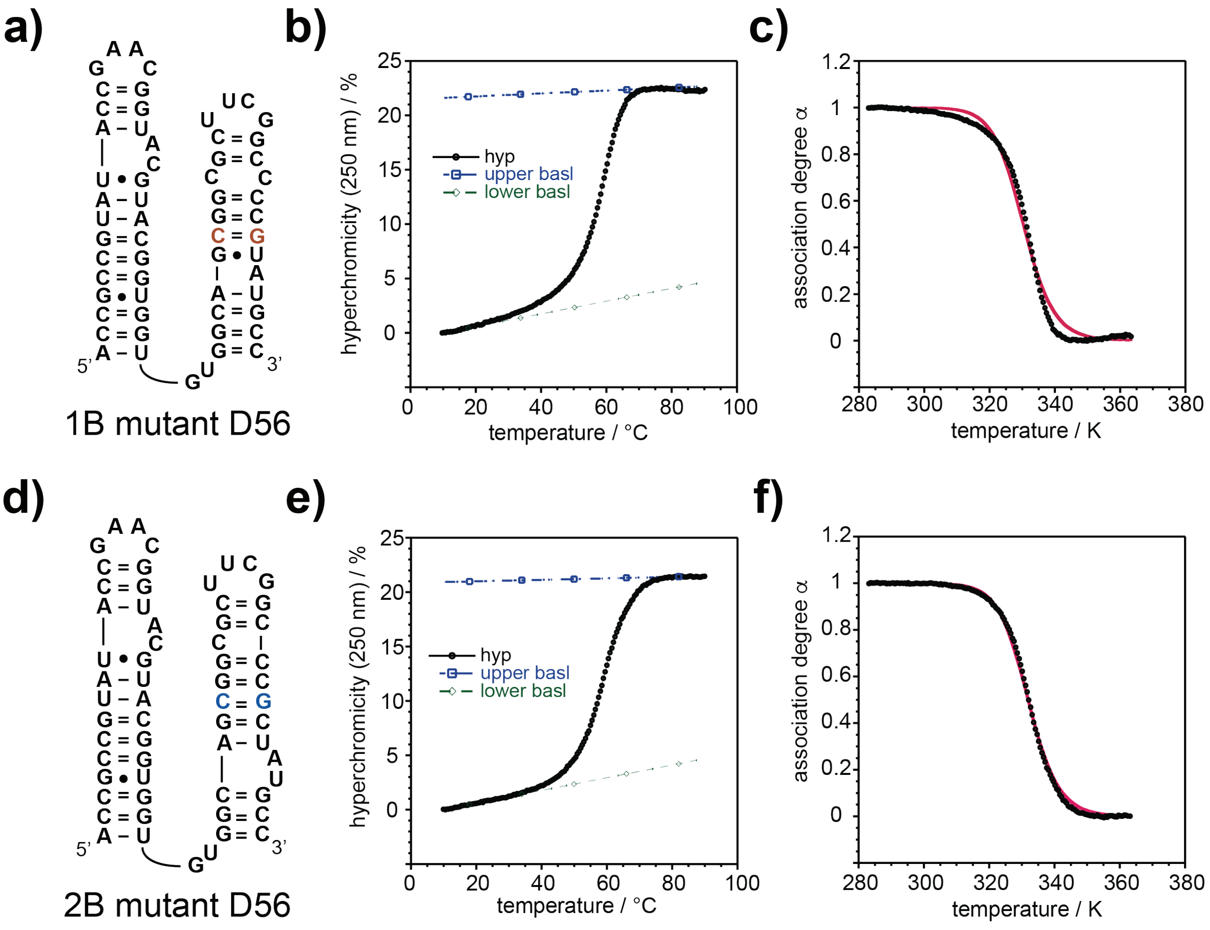

Supplementary Figure 14. Extraction of thermodynamic parameters from UV melting curves. **a)** Secondary structures of 1B mutant D56 RNA. **b)** UV melting curve at 2 µM strand concentration with upper and lower baselines from linear regression. **c)** Association degree a as a function of temperature. Best fit to the equation given in the main manuscript yielding the thermodynamic parameters is shown in red. **d)** Secondary structures of 2B mutant D56 RNA. **e)** UV melting curve at 2 µM strand concentration with upper and lower baselines from linear regression. **f)** Association degree a as a function of temperature. Best fit to the equation given in the main manuscript yielding the thermodynamic parameters is shown in red.

Supplementary Table 3. Summary of melting points and thermodynamic data for the wildtype D56 RNA, 1B D56 mutant and 2B D56 mutant from UV melting curve experiments.

RNA	concentration / μM	T_m / °C [a]	ΔH / kcal mol^{-1} [b]	ΔS / cal mol^{-1} K^{-1} [b]	ΔG / kcal mol^{-1} [c]
**D56 wildtype 61nt**	2	58 ± 1	n.d	n.d.	n.d.
	5	59 ± 1			
**D56 1B mutant 61nt**	2	59 ± 1	-46.1 ± 6.0	-140 ± 18	-4.4 ± 0.8
	5	59 ± 1			
**D56 2B mutant 61nt**	2	60 ± 1	-47.0 ± 2.4	-142 ± 7	-4.6 ± 0.8
	5	61 ± 1			

[a] melting temperature (mean value from five heating/cooling curves) in °C; [b] thermodynamic parameters of the monomolecular melting process were obtained by plotting the association degree alpha versus temperature and fitting the data in *KaleidaGraph* (Synergy Software) according to the equation given in the Methods section; [c] free energy calculated for 298 K. n.d. not determinable.

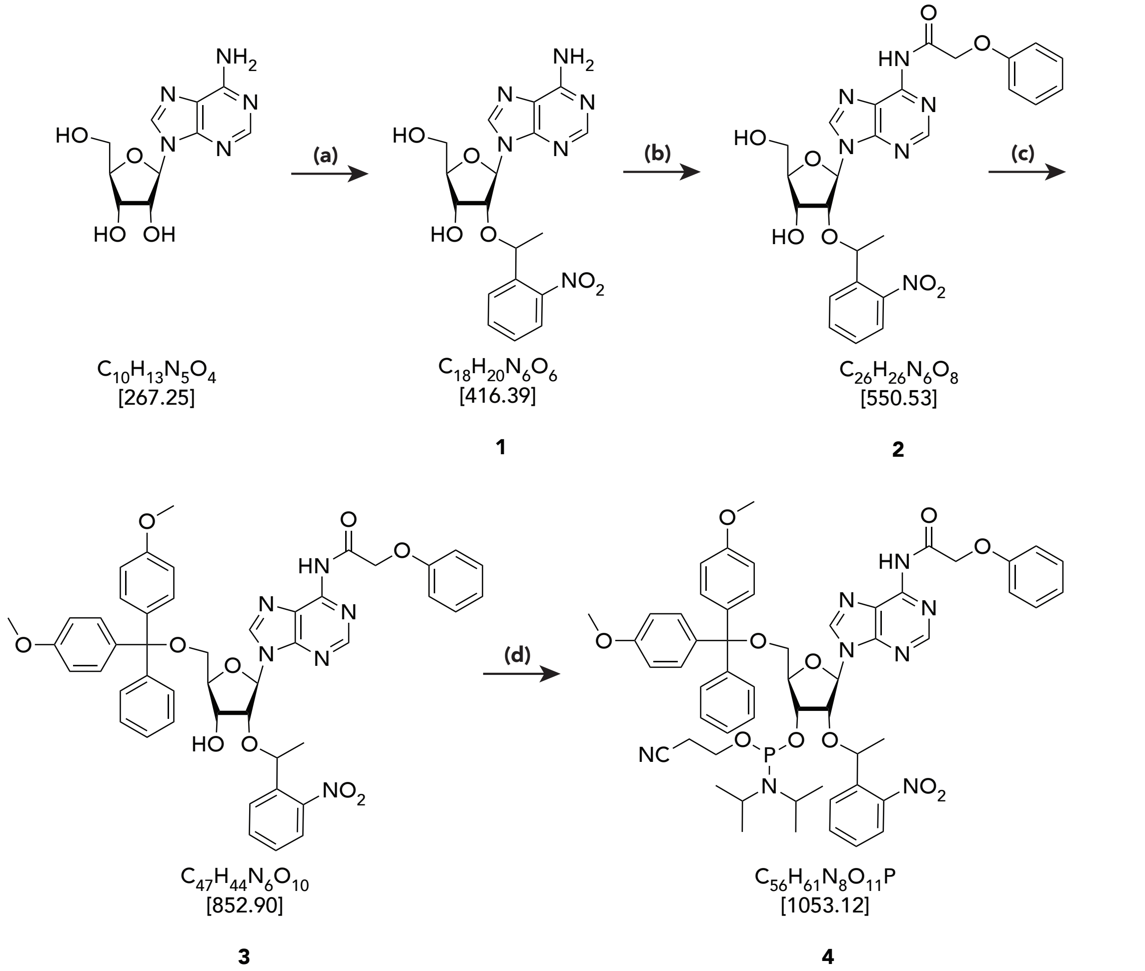

Supplementary Scheme 1. Chemical synthesis of 2'-*O*-photolabile protected adenosine building block **4**. **(a)** NaH, BENB, in DMF, 0°C to rt, 15h, 53%; **(b)** TMS-Cl, Pac-Cl, 1,2,4-triazole, NH_4OH, in pyridine, 0°C to 55°C, 20h, 50%; **(c)** DMT-Cl in pyridine, rt, 2h, 68%; **(d)** CEP-Cl, DIPEA, in CH_2Cl_2, rt, 2.5h, 81%.

Detailed description for the preparation of the 2'-*O*-NPE protected adenosine building block 4.

1. *2'-O- α-Methyl-o-nitrophenyl adenosine*

A solution of commercially available adenosine (2.00 g, 7.48 mmol, 1.00 eq.) in anhydrous *N,N*-dimethylformamide (25 mL) was cooled to 0°C, sodium hydride (296 mg, 11.2 mmol, 1.50 eq.) was added and the solution was stirred under argon atmosphere for one hour. 1-(1-Bromoethyl)-2-nitrobenezene (2.51 g, 10.9 mmol, 1.46 eq.) was added and the mixture was allowed to stir for another 5 hours under equal conditions until TLC confirmed complete conversion. The reaction was quenched by addition of anhydrous ethanol and neutralized with 1 N hydrochloric acid before all solvents were removed. After purification by column chromatography (50 g SiO_2, methylene chloride/ methanol: 100/0 – 96/4) a mixture of 2' and 3' substituted products was obtained as a yellow solid.

Yield: 1.65 g of a yellow solid (3.96 mmol, 53%)
TLC: (Methylene chloride/ methanol: 9/1); R_f = 0.34
^{1}H-NMR (300 MHz, DMSO-d_6, 25°C): 8.32-8.16 (s, 1H, C(8)*H*); 8.00-7.91 (s, 1H, C(2)*H*); 7.93-7.88 (m, 2H, -NO_2C-C*H*(m)-); 7.78-7.70 (m, 1H -NO_2C-C-C*H*(o)-); 7.50-7.54 (m, 1H, -NO_2C-CH-C*H*(p)-,); 7.31-7.21 (m, 3H, -NO_2C-CH-CH-C*H*(m)-, N^6H_2); 6.07-5.93 (m, 1H, C(1')*H*); 5.38-5.03 (m, 4H, C(2')O*H*, C(3')O*H,* C(5')O*H*, -O-C*H*-CH_3-); 4.32-4.24 (m, 1H, C(2')*H*); 4.17-4.14 (m, 1H, C(3')*H*); 4.09-4.03 (m, 1H, C(4')*H*); 3.71-3.47 (m, 1H, C(5')H_2); 1.42-1.38 (d, 3H, -CH-CH_3, $^3J_{HH}$=6.22 Hz)
^{13}C-NMR (75 MHz, DMSO-d_6, 25°C): 153.02 (s, 1C, *C*(6)); 152.95 (s 1C, *C*(2)); 149.65 (s, 1C, *C*(4)); 140.66 (s, 1C, -*C*-NO_2) 139.78 (s, 1C, *C*(8)); 139.95 (s, 1C, -*C*-CNO_2-); 134.61 (s, 1C, -NO_2C-C-*C*H(o)-); 129.28 (s, 1C, -NO_2C-CH-CH-*C*H(m)-); 128.67 (s, 1C, -NO_2C-C-*C*H(o)-); 124.93 (s,1C, -NO_2C-*C*H(m)-); 120.26 (s, 1C, *C*(5)); 87.57 (s, 1C, *C*(1')); 85.95 (s, 1C, *C*(4')); 79.99 (s, 1C, *C*(2')); 72.39 (s, 1C, -O-*C*H-CH_3); 69.28 (s, 1C, *C*(3')); 61.68 (s, 1C, *C*(5')); 24.47 (s, 1C, -CH-CH_3)
ESI-MS: calc. m/z: 416.3940; found: 417.0906 [M+H$^+$]

2. *N^6-Phenoxyacetyl-2'-O-α-methyl-o-nitrophenyl adenosine*

A solution of compound **1** (1.65 g, 3.96 mmol, 1.00 eq.) in anhydrous pyridine (35 mL) was cooled to 0°C, trimethylsilyl chloride (4.07 mL, 32.1mmol, 8.10 eq.) was added and the solution was allowed to warm to room temperature and stirred under argon atmosphere for 45 minutes. A mixture of 1,2,4-triazole (413 mg, 5.97 mmol, 1.51 eq.) and phenoxyacetyl chloride (825 µL, 5.97 mmol, 1.51 eq.) in pyridine/ acetonitrile (1/1, 30 mL) was prepared and slowly added to the reaction before it was heated to 55°C and stirred for 19 hours. After TLC confirmed complete conversion, water (4 mL) was added to quench the reaction. The mixture was cooled to 0°C, 30% aqueous ammonium hydroxide (2.66 mL) was added and stirred for another 30 minutes until all solvents were evaporated. The residual yellow solid was dissolved in methylene chloride and subsequently washed with 5% aqueous citric acid and saturated sodium bicarbonate solution. The aqueous phases were extracted twice with methylene chloride, the organic layers were combined, dried over sodium sulfate and the solvent was evaporated. After purification via column chromatography (50 g SiO_2, ethyl acetate/ n-hexane: 1/1 – 1/0) pure compound **2** was obtained as an off-white foam.

Yield: 1.08 g of an off-white foam (1.96 mmol, 50%)
TLC: (ethyl acetate); R_f = 0.31
^{1}H-NMR (300 MHz, DMSO-d_6, 25°C): 10.88 (s, 1H, N^6*H*); 8.67 (s, 1H, C(8)*H*); 8.52 (s, 1H, C(2)*H*); 7.92 (m, 2H, -NO_2C-C*H*(m)-, -NO_2C-C-C*H*(o)-); 7.72 (t, 1H, -NO_2C-CH-C*H*(p)-,

$^3J_{HH}$=7.50 Hz); 7.48 (t, 1H, -NO_2C-CH-CH-C*H*(m)-, $^3J_{HH}$=7.96 Hz); 7.27 (m, 2H, arom.*H* Pac,m); 6.93 (m, 3H, arom.*H* Pac, o/p); 6.22 (d, 1H, C(1')*H*, $^3J_{HH}$=2.98 Hz); 5.37 (m, 2H, C(3')O*H*, -O-C*H*-CH_3-); 5.09 (t, 1H, C(5')O*H*, $^3J_{HH}$=5.27 Hz) 5.01 (s, 2H, -CO-CH_2-O-); 4.15 (m, 1H, C(2')*H*); 4.09 (m, 1H, C(3')*H*); 4.00 (m, 1H, C(4')*H*); 3.73 (m, 1H, C(5')*H'*); 3.55 (m, 1H, C(5')*H''*); 1.45 (d, 3H, -CH-CH_3, $^3J_{HH}$=6.22 Hz)
<u>^{13}C-NMR (75 MHz, DMSO-d_6, 25°C):</u> 168.27 (s, 1C, -NH-*C*O-CH_2-); 158.99 (s, 1C, -$(CH)_2$-*C*O-); 152.26 (s, 1C, *C*(2)); 151.88 (s, 2C, *C*(4), *C*(6));148.67 (s, 1C, -C-*C*-NO_2-);142.62 (s, 1C C(8)); 139.43 (s, 1C, -*C*-CNO_2-);134.66 (s, 1C, -NO_2C-C-*C*H(o)-); 130.37 (s, 2C, arom.*C* Pac,m); 129.31 (s, 1C, -NO_2C-CH-CH-*C*H(m)-); 128.80 (s, 1C, -NO_2C-C-*C*H(o)-); 125.03 (s,1C, -NO_2C-*C*H(m)-); 124.31 (s, 1C, C(5)); 121.83 (s, 1C, arom.*C* Pac, p); 115.05 (s, 2C, arom.*C* Pac, o), 87.73 (s, 1C, *C*(1')); 85.66 (s, 1C, *C*(4')) 80.18 (s, 1C, *C*(2')); 72.72 (s, 1C, -O-*C*H-CH_3-); 69.01 (s, 1C, *C*(3')); 67.88 (s, 2C, -CO-*C*H_2-O-); 60.85 (s, 1C, *C*(5')); 24.48 (s, 1C, -CH-*C*H_3)
<u>ESI-MS:</u> calc. m/z: 550.5280; found: 551.1931 [M+H^+]

3. *N^6-Phenoxyacetyl-2'-O-α-methyl-o-nitrobenzene-5'-O-(4,4'-dimethoxytrity) adenosine*

To a solution of compound **2** (1.08 g, 1.96 mmol, 1.00 eq.) in anhydrous pyridine (12 mL) 4,4'-dimethoxytrityl chloride (787 mg, 2.35 mmol, 1.20 eq.) was added in three portions and the solution was stirred under argon atmosphere for 2 hours. After TLC confirmed complete conversion methanol (2 mL) was added to quench the reaction. All solvents were evaporated and the residual orange solid was dissolved in methylene chloride and subsequently washed with 5% aqueous citric acid and saturated sodium bicarbonate solution. The organic phase was dried over sodium sulfate, the solvent was evaporated and the crude product was dried *in vacuo* for 30 minutes. After purification via column chromatography (50 g SiO_2, ethyl acetate/ n-hexane: 4/6 – 7/3) pure compound **3** was obtained as a white foam.

<u>Yield:</u> 1.14 g of a white foam (1.34 mmol, 68%)
<u>TLC:</u> (ethyl acetate/ n-hexane: 7/3); R_f = 0.48
<u>^{1}H-NMR (300 MHz, DMSO-d_6, 25°C):</u> 10.91 (s, 1H, N^6*H*); 8.48 (s, 1H, C(2)*H*); 8.45 (s, 1H, C(8)*H*); 7.94 (d, 1H, -NO_2C-C*H*(m)-, $^3J_{HH}$=15.55 Hz); 7.92 (d, 1H, -NO_2C-C-C*H*(o)-, $^3J_{HH}$=15.94 Hz); 7.73 (t, 1H, -NO_2C-CH-C*H*(p)-, $^3J_{HH}$=7.52 Hz); 7.48 (t, 1H, -NO_2C-CH-CH-C*H*(m)-, $^3J_{HH}$=7.67 Hz); 7.30 (m, 2H, arom.*H* Pac,m); 7.19 (m, 9H, arom.*H* DMT); 6.93 (m, 3H, arom.*H* Pac, o/p); 6.79 (m, 4H, DMT, CH_3-O-C-C*H*-); 6.26 (d, 1H, C(1')*H*, $^3J_{HH}$=2.37 Hz); 5.37 (d, 1H, C(3')O*H*, $^3J_{HH}$=6.60 Hz); 5.29 (q, 1H, -O-C*H*-CH_3-, $^3J_{HH}$=5.97 Hz); 5.00 (s, 2H, -CO-CH_2-O-); 4.34 (m, 1H, C(2')*H*); 4.29 (m, 1H, C(3')*H*); 4.14 (m, 1H, C(4')*H*); 3.69 (s, 6H, 2x -O-CH_3), 3.21 (m, 2H, C(5')H_2); 1.96 (s, 3H, -CH-CH_3)

<u>^{13}C-NMR (75 MHz, DMSO-d$_6$, 25°C):</u> 168.15 (s, 1C, -NH-*C*O-CH$_2$-); 159.33 (s, 2C, 2x-*C*-OCH$_3$) 158.67 (s, 1C, -*C*O-, Pac); 152.24 (s, 1C, *C*(2)); 151.89 (s, 2C, *C*(6)); 149.91 (s, 1C, *C*(4)); 148.67 (s, 1C, -C-*C*-NO$_2$-);142.23 (s, 1C C(8)); 139.51 (s, 1C, -*C*-CNO$_2$-);134.59 (s, 1C, -NO$_2$C-C-*C*H(o)-); 133.57-128.01 (m, 16C, 2x arom.*C* Pac,m, 12x arom.*C*, DMT, -NO$_2$C-CH-CH-*C*H(m)-, -NO$_2$C-C-*C*H(o)-); 124.39 (s,1C, -NO$_2$C-*C*H(m)-); 124.31 (s, 1C, C(5)); 122.73 (s, 1C, arom.*C* Pac, p); 115.67 (s, 2C, arom.*C* Pac, o); 114.22 (s, 4C, DMT, CH$_3$-O-C-*C*H-); 89.15 (s, 1C, *C*(1')); 86.58 (s, 1H, -O-*C*-Phe$_3$); 83.46 (s, 1C, *C*(4')); 79.37 (s, 1C, *C*(2')); 73.37 (s, 1C, -O-*C*H-CH$_3$-); 69.68 (s, 1C, *C*(3')); 68.81 (s, 2C, -CO-*C*H$_2$-O-); 63.53 (s, 1C, *C*(5')); 24.09 (s, 1C, -CH-*C*H$_3$)
<u>ESI-MS:</u> calc. m/z: 852.9010; found: 853.3157 [M+H$^+$], 875.3204 [M+Na$^+$]

4. *N^6-Phenoxyacetyl-2'-O-α-methyl-o-nitrophenyl-5'-O-(4,4'-dimethoxytrity) adenosine-3'-O-(2-cyanoethyl-N,N-diisopropylphosphoramidite)*

To a solution of compound **3** (810 mg, 0.95 mmol, 1.00 eq.) in anhydrous methylene chloride (6 mL) *N,N*-diisopropylethylamine (724 µL, 4.15 mmol, 4.37 eq.) was added and the solution was stirred under argon atmosphere for 20 minutes. 2-cyanoethyl-N,N-diisopropylchlorophosphoramidite (443 µL, 2.00 mmol, 2.10 eq.) was added and the solution was allowed to stir for another 2 hours. After TLC confirmed complete conversion methanol (2 mL) was added to quench the reaction. The mixture was diluted with methylene chloride and washed with half-saturated sodium bicarbonate solution. The organic phase was dried over sodium sulfate, the solvent was evaporated and the crude product was shortly dried *in vacuo* before it was purified via column chromatography (50 g SiO_2, ethyl acetate/ n-hexane: 2/8 – 7/3 + 1% triethylamine) to give pure compound **3** as a white foam consisting of two diastereomeres.

<u>Yield:</u> 810 mg of a white foam (0.77 mmol, 81%)
<u>TLC:</u> (ethyl acetate/ n-hexane: 1/1); R_f = 0.52 + 0.57
<u>^{1}H-NMR (300 MHz, CDCl$_3$, 25°C):</u> 9.40 (s, 1H, N^6*H*); 8.68 (s, 1H, C(2)*H*); 8.25 (s, 1H, C(8)*H*); 7.87 (m, 2H, -NO$_2$C-C*H*(m), -NO$_2$C-C-C*H*(o)); 7.62 (m, 1H, -NO$_2$C-CH-C*H*(p)); 7.42-7.21 (m, 13H, -NO$_2$C-CH-CH-C*H*(m), arom.*H* (Pac,m), arom.*H* (DMT)); 7.06 (m, 3H, arom.*H* Pac, o/p); 6.80 (m, 4H, DMT, CH$_3$-O-C-C*H*-); 6.22 (m, 1H, C(1')*H*); 5.35 (m, 1H, -O-C*H*-CH$_3$-); 4.89 (s, 2H, -CO-C*H$_2$*-O-); 4.75 (m, 1H, C(2')*H*); 4.47 (m, 1H, C(3')*H*); 4.38 (m, 1H, C(4')*H*); 3.78 (s, 6H, 2x -O-C*H$_3$*), 3.64-3.30 (m, 6H, C(5')*H$_2$*, -C*H$_2$*-C*H$_2$*-CN); 2.41 (m, 2H, 2x -N-C*H*-(CH$_3$)$_2$); 1.49 (m, 3H, -O-CH-C*H$_3$*); 1.11-0.93 (m, 12H, 2x -N-CH-(C*H$_3$*)$_2$);
<u>^{13}C-NMR (75 MHz, CDCl$_3$, 25°C):</u> 167.04 (s, 1C, -NH-*C*O-CH$_2$-); 159.02 (s, 2C, 2x-*C*-OCH$_3$) 157.45 (s, 1C, -*C*O-, Pac); 153.11 (s, 1C, *C*(2)); 151.81 (s, 2C, *C*(4), *C*(6));148.14 (s, 1C, -C-*C*-NO$_2$-);142.42 (s, 1C C(8)); 139.47 (s, 1C, -*C*-CNO$_2$-); 133.93 (s, 1C, -NO$_2$C-C-*C*H(o)-);

130.61-127.49 (m, 16C, 2x arom.*C* Pac,m, 12x arom.*C*, DMT, -NO_2C-CH-CH-*C*H(m)-, -NO_2C-C-*C*H(o)-); 124.66 (s,1C, -NO_2C-*C*H(m)-); 123.81 (s, 1C, C(5)); 122.75 (s, 1C, arom.*C* Pac, p); 117.97 (s, 1C, -CH_2-*C*N) 115.47 (s, 2C, arom.*C* Pac, o); 113.47 (s, 4C, DMT, CH_3-O-C-*C*H-); 88.01 (s, 1C, *C*(1')); 87.11 (s, 1H, -O-*C*-Phe_3); 83.98 (s, 1C, *C*(4')); 78.69 (s, 1C, *C*(2')); 73.06 (s, 1C, -O-*C*H-CH_3-); 71.44 (s, 1C, *C*(3')); 63.33 (s, 1C, *C*(5')); 58.58 (s, 1C, -P-O-*C*H_2-CH_2-); 55.72 (s, 2C, -CO-*C*H_2-O-); 43.47 (s, 2C, 2x-N-*C*H-$(CH_3)_2$); 25.23 (s, 4C, 2x-N-CH-(*C*$H_3)_2$); 23.49 (s, 1C, -CH-*C*H_3); 20.35 (s, 1C, -O-CH_2-*C*H_2-CN)
^{31}P-NMR (121 MHz, $CDCl_3$, 25°C): 151.38 (s,1P); 151.21 (s, 1P)
ESI-MS: calc. m/z: 1053.1228; found: 1053.3953 [M+H$^+$],1075.4080 [M+Na$^+$], 1091.3682 [M+K$^+$]
